# Nephronophthisis gene products display RNA-binding properties and are recruited to stress granules

**DOI:** 10.1038/s41598-020-72905-8

**Published:** 2020-09-29

**Authors:** Luisa Estrada Mallarino, Christina Engel, İbrahim Avşar Ilık, Daniel Maticzka, Florian Heyl, Barbara Müller, Toma A. Yakulov, Jörn Dengjel, Rolf Backofen, Asifa Akhtar, Gerd Walz

**Affiliations:** 1 Renal Division, Department of Medicine, University Freiburg Medical Center, Faculty of Medicine, University of Freiburg, Hugstetter Str. 55, 79106 Freiburg, Germany; 2 Faculty of Biology, University of Freiburg, Freiburg, Germany; 3 Max Planck Institute of Immunobiology and Epigenetics, Freiburg, Germany; 4 Institute for Informatics, Albert-Ludwigs-University, Freiburg, Germany; 5 Department of Dermatology, Medical Center, and Freiburg Institute for Advanced Studies, University of Freiburg, Freiburg, Germany; 6 CIBSS-Centre for Integrative Biological Signalling Studies, University of Freiburg, Freiburg, Germany; 7 Signalling Research Centres BIOSS and CIBSS, University of Freiburg, Freiburg, Germany; 8 Present Address: Max Planck Institute for Molecular Genetics, 14195 Berlin, Germany; 9 Present Address: Department of Biology, University of Fribourg, 1700 Fribourg, Switzerland

**Keywords:** Cell biology, Developmental biology, Genetics, Molecular biology, Pathogenesis

## Abstract

Mutations of cilia-associated molecules cause multiple developmental defects that are collectively termed ciliopathies. However, several ciliary proteins, involved in gating access to the cilium, also assume localizations at other cellular sites including the nucleus, where they participate in DNA damage responses to maintain tissue integrity. Molecular insight into how these molecules execute such diverse functions remains limited. A mass spectrometry screen for ANKS6-interacting proteins suggested an involvement of ANKS6 in RNA processing and/or binding. Comparing the RNA-binding properties of the known RNA-binding protein BICC1 with the three ankyrin-repeat proteins ANKS3, ANKS6 (NPHP16) and INVERSIN (NPHP2) confirmed that certain nephronophthisis (NPH) family members can interact with RNA molecules. We also observed that BICC1 and INVERSIN associate with stress granules in response to translational inhibition. Furthermore, BICC1 recruits ANKS3 and ANKS6 into TIA-1-positive stress granules after exposure to hippuristanol. Our findings uncover a novel function of NPH family members, and provide further evidence that NPH family members together with BICC1 are involved in stress responses to maintain tissue and organ integrity.

## Introduction

Nephronophthisis (NPH) belongs to a group of autosomal recessive syndromes associated with cystic kidney disease in combination with other extrarenal manifestations^1^. Most gene products mutated in NPH localize to the primary cilium, a non-motile, microtubule-based organelle attached to almost all mammalian cells^2^. Therefore, the disruption of ciliary structure and/or function is thought to underlie the renal and extrarenal manifestations observed in NPH and related syndromes^3^.

Several NPH gene products (NPHPs) localize to subcellular compartments outside of the cilium, including cell–cell junctions^4^, the secretory pathway^5^ and the nucleus^6^. However, it is unclear whether NPHPs at these sites participate in specific cellular functions independent of the cilium. Recently, several NPHPs with nuclear localization have been implicated in DNA damage responses^7–9^, suggesting that cilia-associated NPHPs participate in developmental programs, while NPHPs outside of the cilium are involved in maintaining cellular homeostasis^10^. Accordingly, defective NPH gene functions may cause both, early onset tissue defects, for example polydactyly at birth as well as more chronic phenotypes such as cystic kidney disease or liver fibrosis that causes end-stage renal failure and liver failure later in life. Curiously, the cyst formation, often limited to the cortico-medullary junctions, is associated with extensive inflammation and interstitial infiltration, supporting the hypothesis that a chronic maladaptation may trigger this manifestation.

To understand the development and homeostatic programs controlled by NPHPs, we compared INVERSIN (NPHP2) and ANKS6 (NPHP16) to the structurally related proteins ANKS3 and BICC1. INVERSIN and ANKS6 share N-terminal ankyrin repeats with ANKS3, while ANKS3 and ANKS6 share the SAM domain with BICC1. All four proteins have been implicated in cystic kidney disease, albeit their molecular functions appear to be quite divergent. INVERSIN localizes to a confined region adjacent to the transient zone of cilia; this part of the ciliary axoneme has therefore been termed INVERSIN segment, and localization of other ciliary proteins to this segment depend on the integrity of INVERSIN function^11,12^. Since INVERSIN also localizes to the plasma membrane and the nucleus^6,13,14^, it has been difficult to correlate localization and molecular functions of INVERSIN with the manifestations caused by INVERSIN mutations. ANKS6 mutations cause NPH-typical manifestations; however, the manifestations caused by ANKS6 also include phenotypes (in particular cardiac malformations) that are less often observed in other types of NPH, but shared with INVERSIN mutations. ANKS6 was identified as a NPHP9/NEK8-interacting protein, and a subsequent screen for ANKS6-interacting proteins identified ANKS3, an ankyrin-repeat protein that likely plays a role in NPH^15–18^.

We observed that NPH members interact with BICC1, the mammalian homologue of the *Drosophila* bicaudal C (BicC)^15^, and detailed structural analysis confirmed the interaction of BICC1 with ANKS3 and ANKS6^19^. Required during *Drosophila* oogenesis to down-regulate *oskar* mRNA at the anterior pole^20^, BicC contains three conserved N-terminal K-homology (KH) domains responsible for RNA binding, and a C-terminal Sterile Alpha Motif domain (SAM), which recruits BICC1 into RNA-processing bodies (P-bodies)^21^. *BicC* binds its own mRNA, and represses its translation by recruiting the CCR4-NOT complex^22^. Deletion of mouse *Bicc1* affects cilia-driven fluid flow at the ventral node, causing randomization of the left–right asymmetry of visceral organs, and it was hypothesized that vertebrate BicC affects ciliary polarization by regulating P-bodies^21^. BicC/BICC1 share the SAM domain with ANKS3 and ANKS6 (Supplementary Fig. 1). SAM domains are typically involved in protein–protein interactions and oligomerization, but have also been implicated in RNA binding^23^, and the recruitment of vertebrate Bicaudal C homologues to P-bodies^21^. Our findings reveal that the three ankyrin repeat-containing proteins ANKS3, ANKS6 and INVERSIN display RNA-binding properties that partially overlap with the profile of RNA molecules interacting with BICC1. Upon translational inhibition imposed by hippuristanol, BICC1 recruited ANKS3 and ANKS6 into stress granules, while INVERSIN assumed this localization independent of BICC1.

## Results

### The NPH family members INVERSIN (NPHP2) and ANKS6 (NPHP16) exhibit RNA-binding properties

To obtain more insight into the molecular functions controlled by ANKS6, we performed a SILAC-based mass spectrometry screen for ANKS6-interacting proteins, using mouse IMCD cells (Supplementary Table 1). GO-term analysis revealed that a large number of enriched ANKS6-interactors were proteins involved in RNA-binding and/or processing (Supplementary Fig. 2). The putative interaction with RNA-binding and -processing proteins raised the question, whether ANKS6 itself is an RNA-binding protein and can associate with distinct RNA molecules.

**Figure 1:**
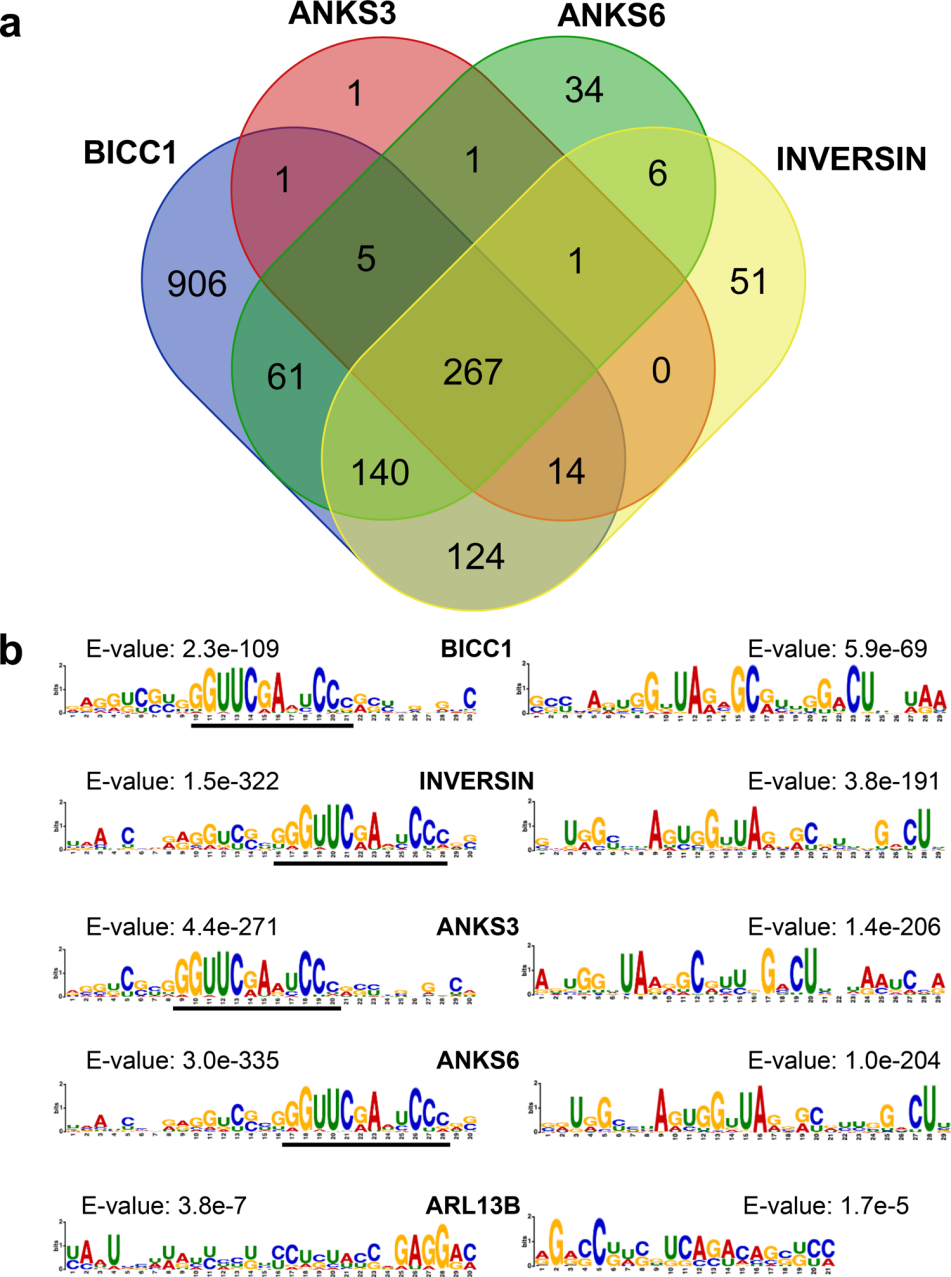
RNA interaction with BICC1 and the ankyrin-repeat proteins ANKS3, ANKS6 and INVERSIN. (**a**) Venn diagram (www.bioinformatics.psb.urgent.be/webtools/Venn/), representing the overlap between RNA molecules interacting with BICC1, ANKS3, ANKS6 and INVERSIN. (**b**) The top two binding motifs for BICC1, INVERSIN, ANKS3, and ANKS6. Two putative binding motifs were also identified for ARL13B, albeit with much lower statistical significance. No significant binding motif (p < 0.05) was identified for BBS3.

**Figure 2:**
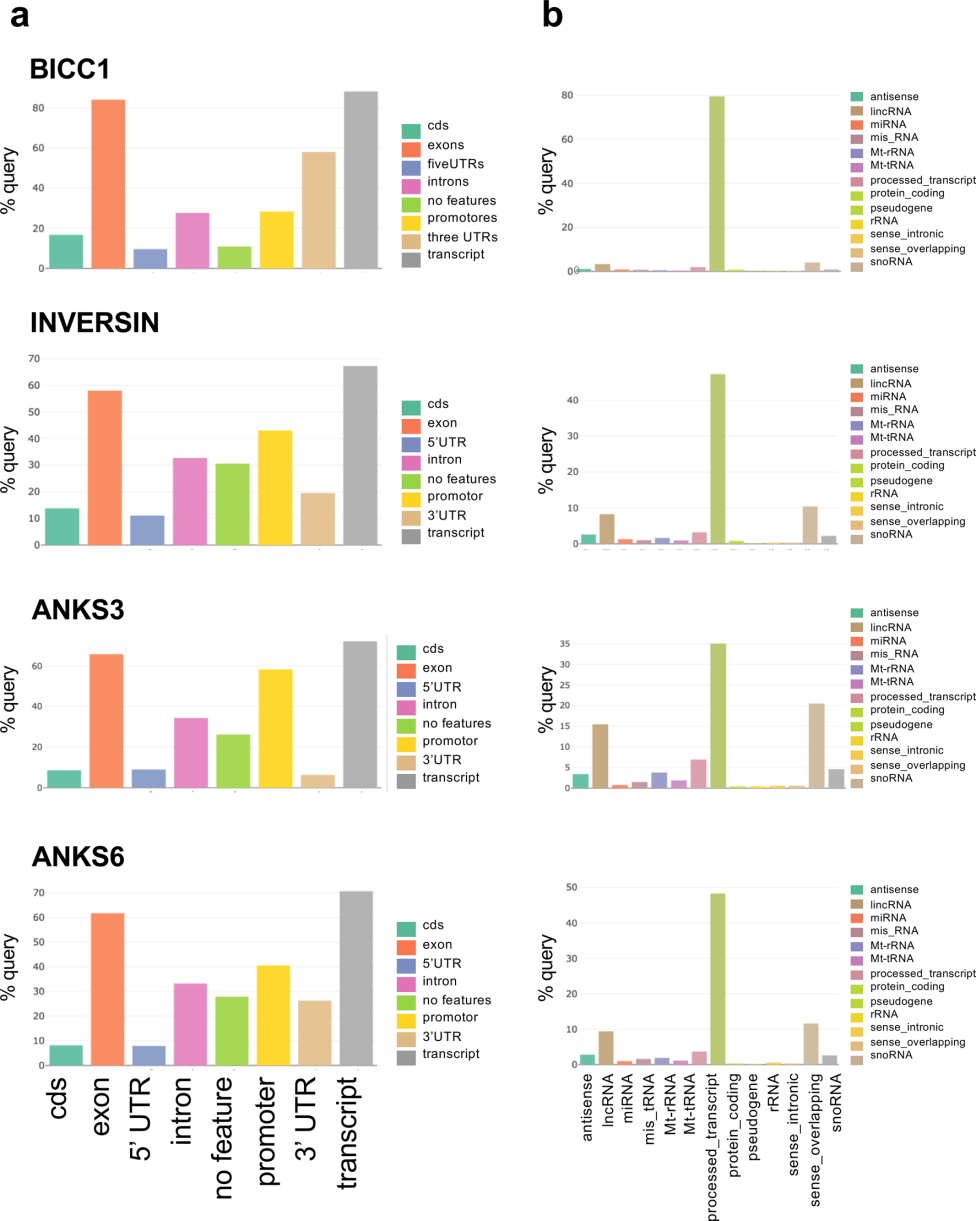
RNA-binding properties of BICC1, ANKS3, ANKS6 and INVERSIN. (**a**) Depicted is the distribution of query regions with certain gene features. All four proteins recognize exons in transcripts. (**b**) Depicted is the distribution of query regions in the genome grouped by gene type. All four proteins recognize protein-coding regions. The analysis was performed with the RNA Centric Analysis System (RCAS) report tool on Galaxy (BIMSB, Max-Delbrück Center for Molecular Medicine, Berlin).

Since ANKS6 shares the ankyrin repeats with ANKS3 and INVERSIN, and the SAM domain with BICC1, we compared the RNA-binding properties of ANKS6, with INVERSIN, ANKS3 and BICC1 by FLASH, an UV light-based method that links RNA to interacting proteins in Flp-in cell lines stably expressing Flag-tagged proteins of interest (Supplementary Fig. 3a)^24^. We included BICC1 in our analysis, since BICC1 is an established RNA-binding protein, and used two cilia-associated molecules without known RNA-binding domains, ARL13B and BBS3, as negative controls. Since ANKS3 and ANKS6 are structurally related, we hypothesized that they may exert similar binding properties. While only few RNA molecules interacted with either ARL13B or BBS3, likely representing non-specific interactions, this approach identified a total of 1518 RNA molecules for BICC1, 603 RNA molecules for INVERSIN, 524 RNA molecules for ANKS6, and 290 RNA molecules for ANKS3 not found in the ARL13B and BBS3 controls. To obtain a general overview between the replicates, we calculated the Pearson correlation coefficients on the de-duplicated files and plotted a heatmap (Supplementary Fig. 3b). The BICC1 samples showed very strong correlation ranging between 0.788 and 0.96, whereas the other proteins showed a moderate to strong correlation (0.478–0.96 for the ANKS6 samples, 0.44–0.972 for the ANKS3 samples and 0.432–0.938 for the INVS samples). The correlation of the negative controls likely resulted from binding abundant, non-specific RNAs (0.661–0.899 for the ARL13B samples and 0.275–0.575 for the BBS3 samples). The Pearson correlation revealed a closer relation between BICC1, ANKS6 and INVS than to ANKS3; similar results were observed in the Venn diagram (Fig. 1a), suggesting similar biological functions between BICC1, ANKS6 and INVS. A total of 267 RNAs bound to all four proteins (Fig. 1a and Supplementary Table 2). Analysis of the top-ranked RNA binding motif for each protein revealed a high degree of similarity with a shared GGUUCRANYCC motif present in all four proteins (Fig. 1b, and Supplementary Fig. 4)^25^. While only two low-scoring motifs without overlap were recorded for ARL13B (Fig. 1b), no specific motifs were identified for BBS3. Regarding the depth of coverage of query regions around the transcriptional start and end sites, all four proteins primarily recognized transcripts within the 5′ end, while BICC1 and INVERSIN also showed a preference for the 3′ end (RNA Centric Analysis System (RCAS) report; https://rna.usegalaxy.eu/). The coverage profile based on the distribution of query regions displayed similarities between BICC1 and ANKS6, and between INVERSIN and ANKS3 (Supplementary Fig. 5), while all four proteins had a preference for exons and protein-coding RNA (Fig. 2). Assigning scores in relationship to their number of binding sites, the top ranked interacting RNA molecules suggested that INV and ANKS3 display closely related binding properties, while ANKS6 partially overlapped with INVERSIN, ANKS3 and BICC1 (Supplementary Fig. 6). GO-term biological analysis of the interacting RNA molecules revealed a preference for mRNA metabolism for BICC1, cytoplasmic translation and organic substance metabolism for INVERSIN and ANKS3, and negative regulation of biological process and mRNA metabolism for ANKS6 (Supplementary Fig. 7a), supporting a function mediated by RNA interaction for all four proteins.

### Interaction of NPHP proteins with *AGO2*

**Figure 3:**
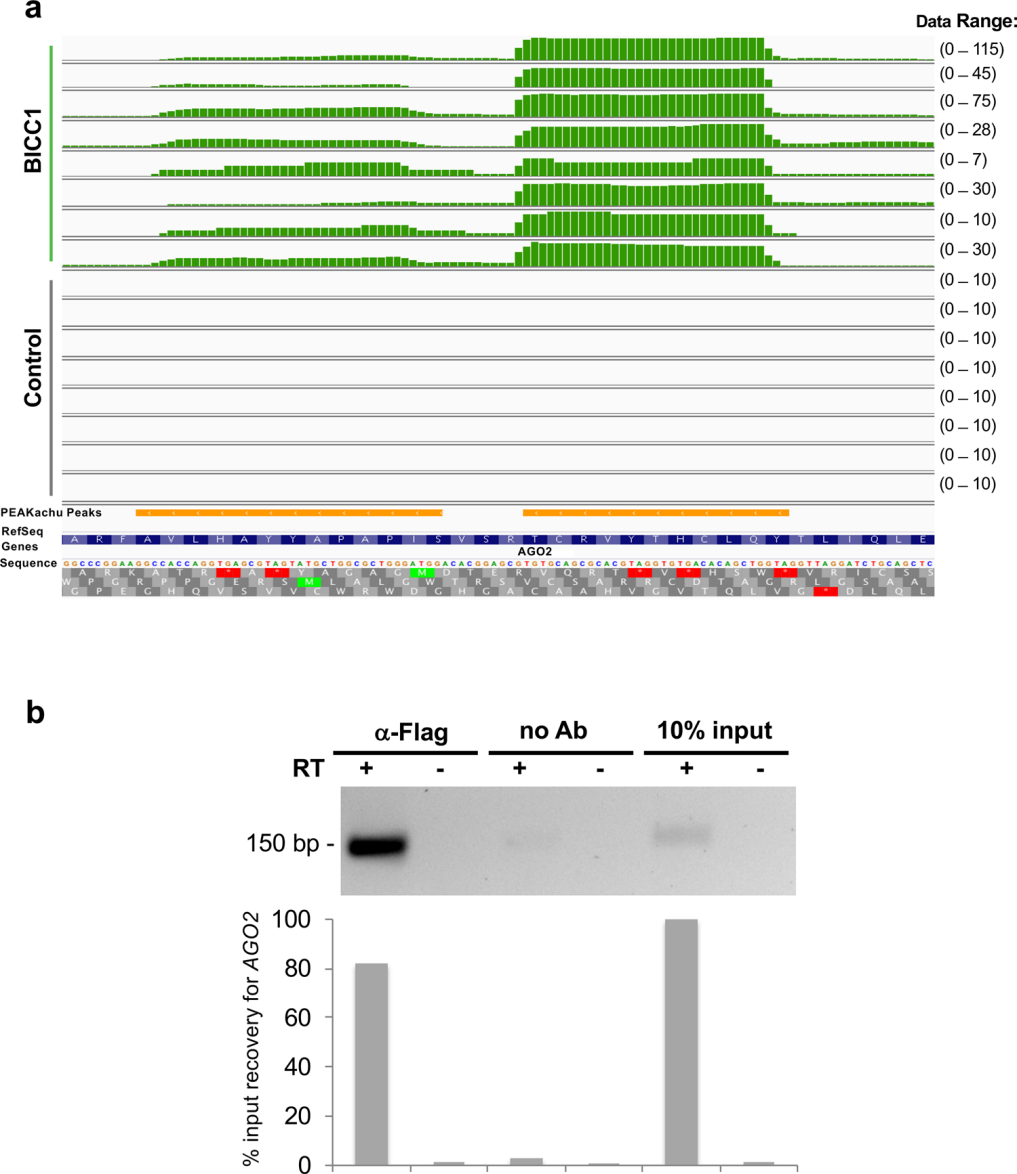
Interaction between BICC1 and *AGO2* mRNA. (**a**) Integrative Genomics Viewer (IGV) snapshot of the FLASH experiments showing FLAG-tagged BICC1 (green) binding on AGO2 exon, the background control (grey) and the position of the PEAKachu results (orange). The coverage of uniquely mapped alignments for each profile is shown in the data range. (**b**) Flag-tagged BICC1 was precipitated, and interacting AGO2 mRNA was reverse-transcribed and amplified by PCR, yielding a 145 bp band. Controls without reverse transcriptase (RT) were essentially negative. In the bar graph, the input was adjusted to 100%. The RNA-precipitation assays were performed three times. The uncropped electrophoresis gel is shown in Supplementary Fig. 13. Alpha Imager V.4.1.0.2 was used for visualization.

Since the FLASH assays identified *AGO2* as one of the BICC1-interacting mRNAs (Fig. 3a), we confirmed this interaction by precipitating BICC1 and analyzing the precipitated RNAs by RT-PCR (Fig. 3b). Since the top BICC1 RNA-binding motifs overlapped with the binding motifs of ANKS3, ANKS6 and INVERSIN, we tested their interaction with *AGO2* mRNA. We found similar binding properties for BICC1 and ANKS6, while only marginal *AGO2* mRNA binding and no binding were detected for ANKS3 and INVERSIN, respectively (Supplementary Fig. 7b). Argonaute proteins associate with miRNAs to repress translation; depending on the cellular state, they accumulate in polyribosomes as well as in various RNA processing bodies, including stress granules^26^. We found that AGO2 and the stress granule (SG) protein CAPRIN1 interacted with all four proteins (Supplementary Fig. 8), supporting the hypothesis that NPH gene products can associate with SGs in response to stress. To further substantiate the relationship between AGO2 and NPH proteins, we utilized the observation that knockdown of zebrafish *anks3* causes cyst formation^17,18^. Cyst formation by depletion of *anks3*, using low concentrations of a splice-blocking morpholino-oligonucleotide (SBM), was significantly augmented by zebrafish *ago2* depletion, (Supplementary Fig. 9), suggesting a genetic interaction between *anks3* and *ago2*.

### Association of BICC1 and INVERSIN in stress granules in response to translational inhibition

**Figure 4:**
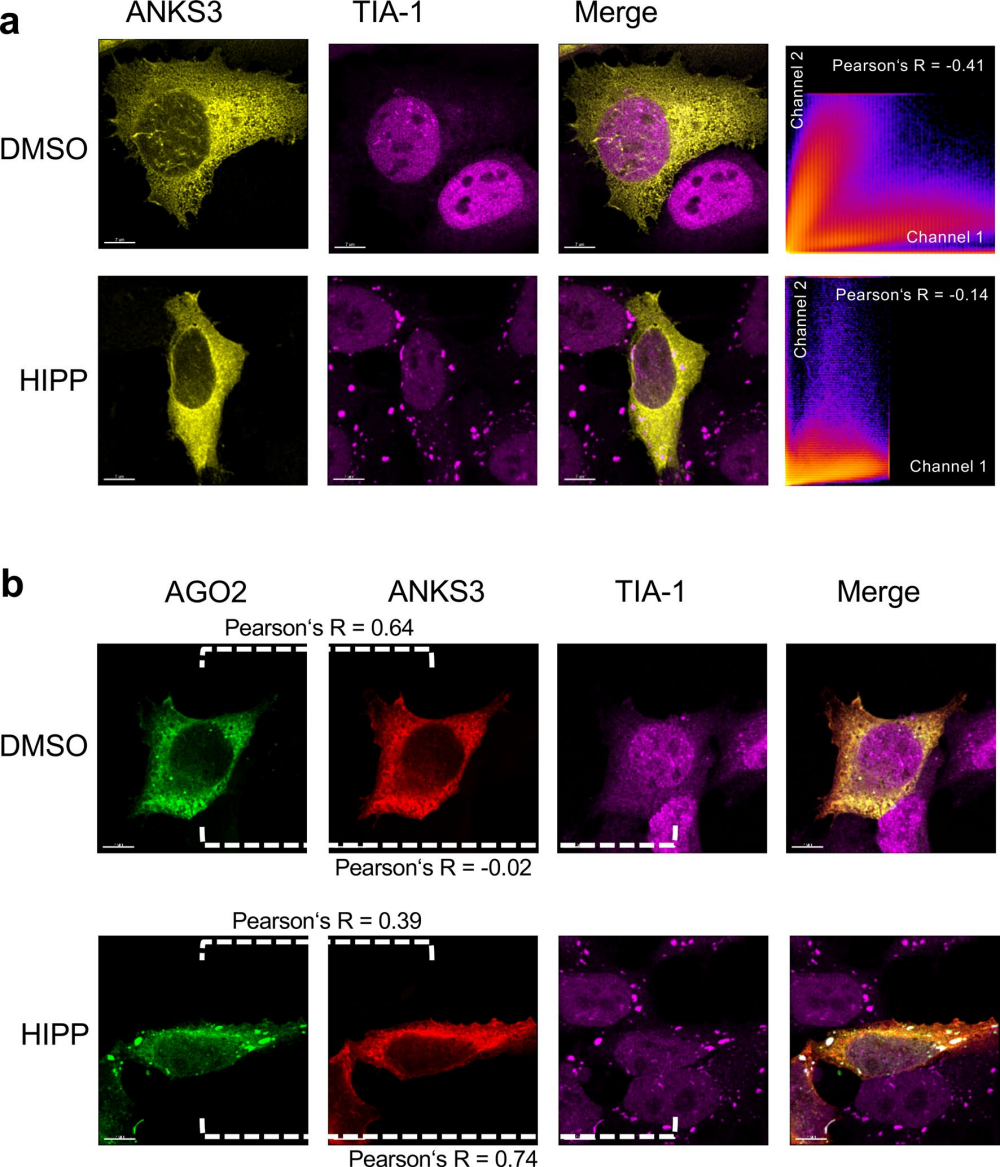
Localization of ANKS3 in response to hippuristanol. (**a**) HeLa cells were transfected with RFP-tagged ANKS3, and either exposed to DMSO or 1 mM hippuristanol for 1 h. TIA-1-positive stress granules formed in response to 1 mM hippuristanol treatment. However, no co-localization was observed between TIA-1-positive stress granules and ANKS3. The experiment was performed twice, analyzing a total of 40 images and 14 z-stacks. (**b**) Co-transfection of GFP-tagged AGO2 or treatment with 1 mM hippuristanol did not alter the localization of ANKS3, while AGO2 partially co-localized with TIA-1-positive stress granules. The experiment was performed twice, analyzing a total of 41 images and 18 z-stacks.

**Figure 5:**
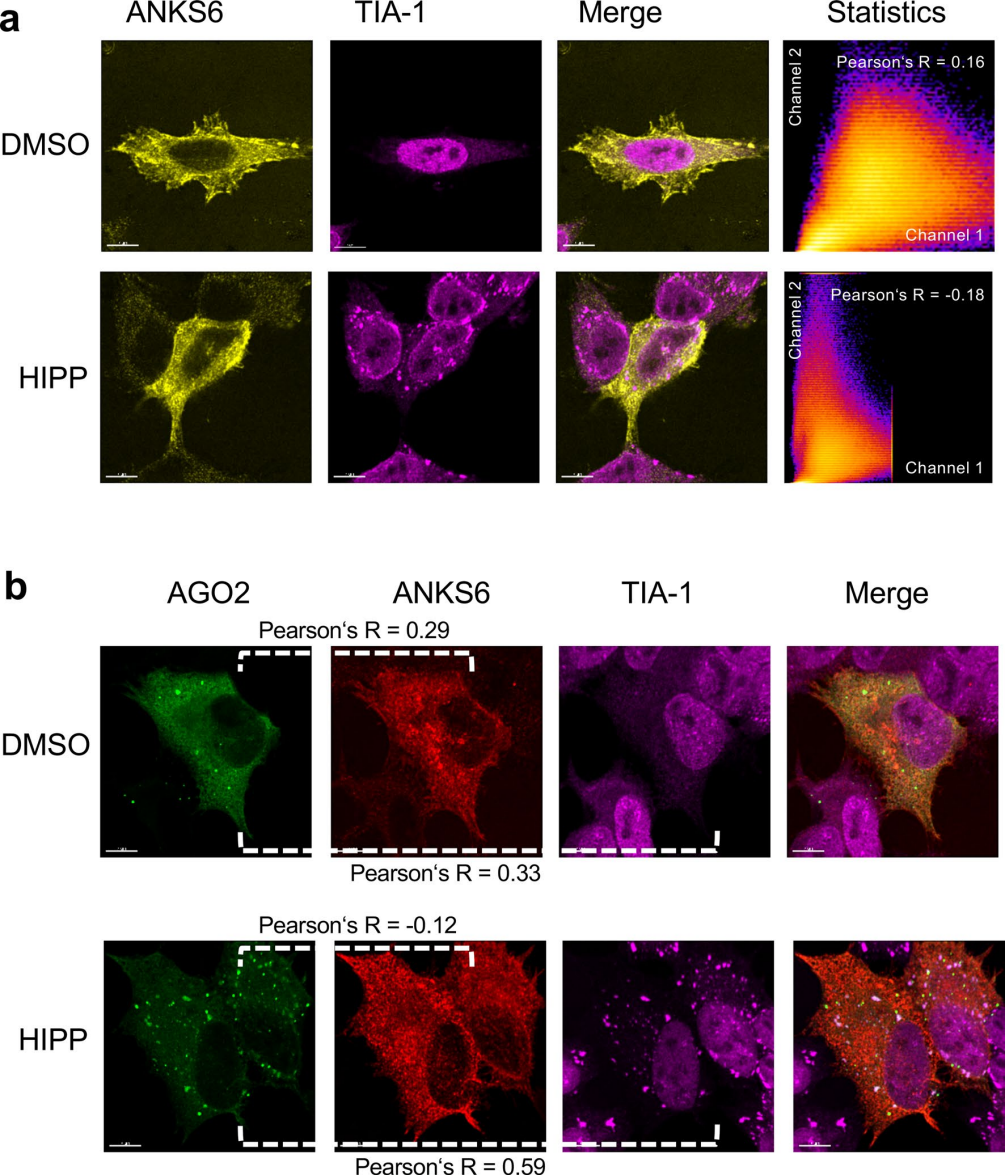
Localization of ANKS6 in response to hippuristanol. (**a**) HeLa cells were transfected with RFP-tagged ANKS6, and either exposed to DMSO or 1 mM hippuristanol for 1 h. TIA-1-positive stress granules formed in response to 1 mM hippuristanol treatment. However, no co-localization was observed between TIA-1-positive stress granules and ANKS6. The experiment was performed three times, analyzing a total of 23 images and 12 z-stacks. (**b**) Co-transfection of GFP-tagged AGO2 (lower two panels) or treatment with 1 mM hippuristanol did not alter the localization of ANKS6, while AGO2 partially co-localized with TIA-1-positive stress granules. The experiment was performed three times, analyzing a total of 33 images and 15 z-stacks.

Mediated by the SAM domain, BICC1 accumulates in cytoplasmic granules that have previously been identified as RNA-processing bodies (P-bodies)^21^ (Supplementary Fig. 10a). In some cells, probably expressing higher levels of BICC1, the BICC1-positive granules co-localized with T-cell Intracellular Antigen-1 (TIA-1), a marker of stress granules. Addition of hippuristanol, a potent steroid inhibitor of the RNA helicase and eukaryotic initiation factor 4A (eIF4A)^27^, resulted in co-localization with TIA-1 and accumulation of BICC1 in stress granules (Supplementary Fig. 10a). In contrast, GFP-tagged AGO2 did not associate with stress granules or TIA-1 in the absence of hippuristanol. However, hippuristanol caused the re-localization of AGO2 into TIA-1-positive granules in response to translational inhibition (Supplementary Fig. 10b). ANKS3, tagged with RFP, assumed a cytoplasmic localization that did not change in response to hippuristanol treatment, or co-transfection of AGO2-GFP (Fig. 4). Although the staining pattern of ANKS6 appeared more granular after treatment with hippuristanol, there was also no overlap with AGO2 or TIA-1 after hippuristanol-induced stress (Fig. 5).

### Recruitment of ANKS3 and ANKS6 into stress granules by BICC1

**Figure 6:**
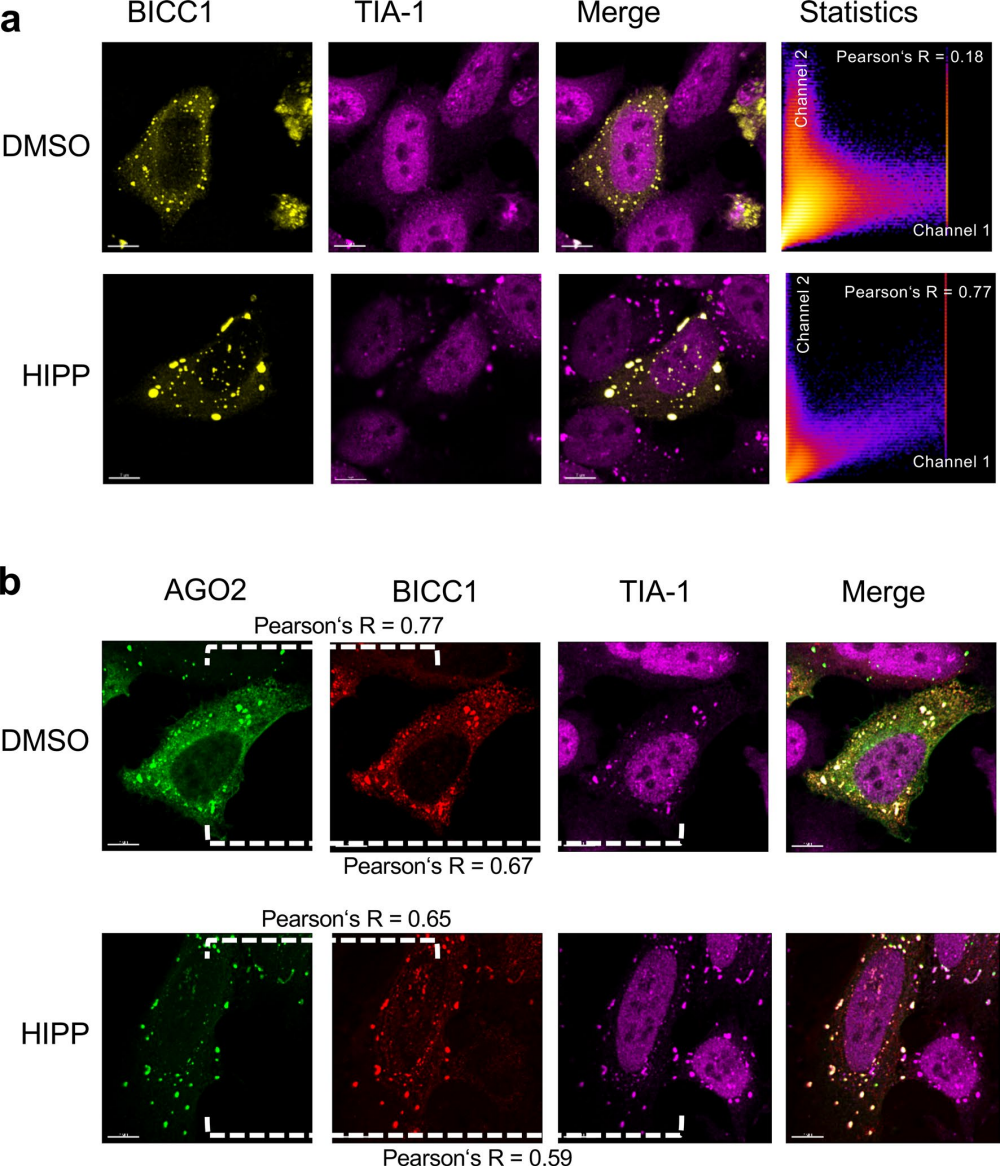
Localization of BICC1 in response to hippuristanol. (**a**) HeLa cells were transfected with RFP-tagged BICC1, and either exposed to DMSO or 1 mM hippuristanol for 1 h. BICC1 accumulated in granules without co-localization with TIA-1 in the absence of stress. Addition of hippuristanol recruited BICC1 into TIA-1-positive stress granules. The experiment was performed four times, analyzing a total of 58 images and 15 z-stacks. (**b**) Co-transfection of GFP-AGO2 caused partial co-localization of BICC1 and AGO2 with TIA-1-positive granules. Hippuristanol exposure recruited most BICC1/AGO2-granules into TIA-1-positive stress granules. The experiment was performed four times, analyzing a total of 91 images and 25 z-stacks.

**Figure 7:**
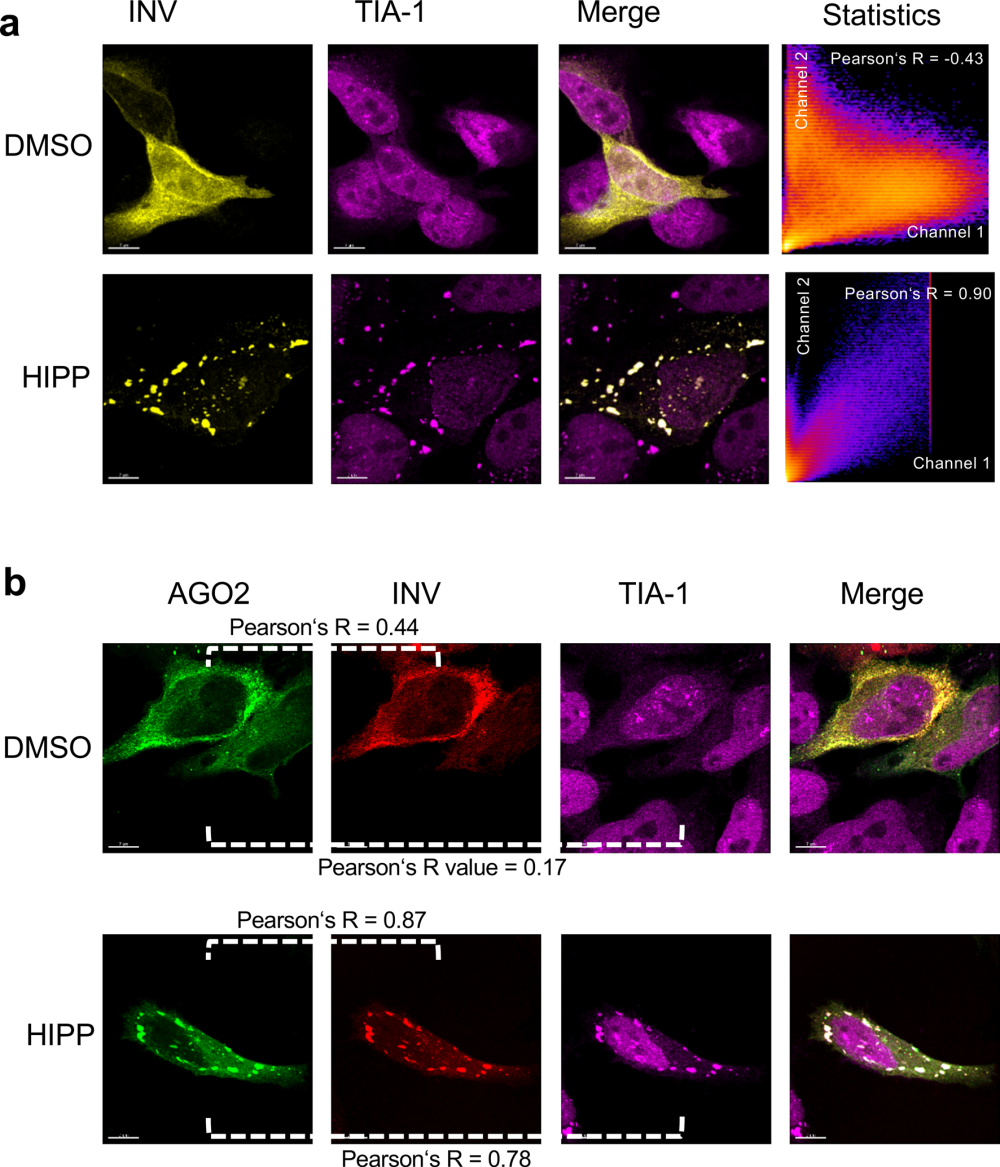
Localization of INVERSIN in response to hippuristanol. (**a**) HeLa cells were transfected with RFP-tagged INVERSIN, and either exposed to DMSO or 1 mM hippuristanol for 1 h. INVERSIN assumed a diffuse cellular localization. Upon treatment with 1 mM hippuristanol, INVERSIN accumulated in TIA-1-positive stress granules. The experiment was performed four times, analyzing a total of 72 images and 22 z-stacks. (**b**) Co-transfection of GFP-tagged AGO2 did not alter the localization of INVERSIN. However, hippuristanol exposure recruited AGO2/INVERSIN-granules into TIA-1-positive stress granules. The experiment was performed five times, analyzing a total of 67 images and 24 z-stacks.

**Figure 8:**
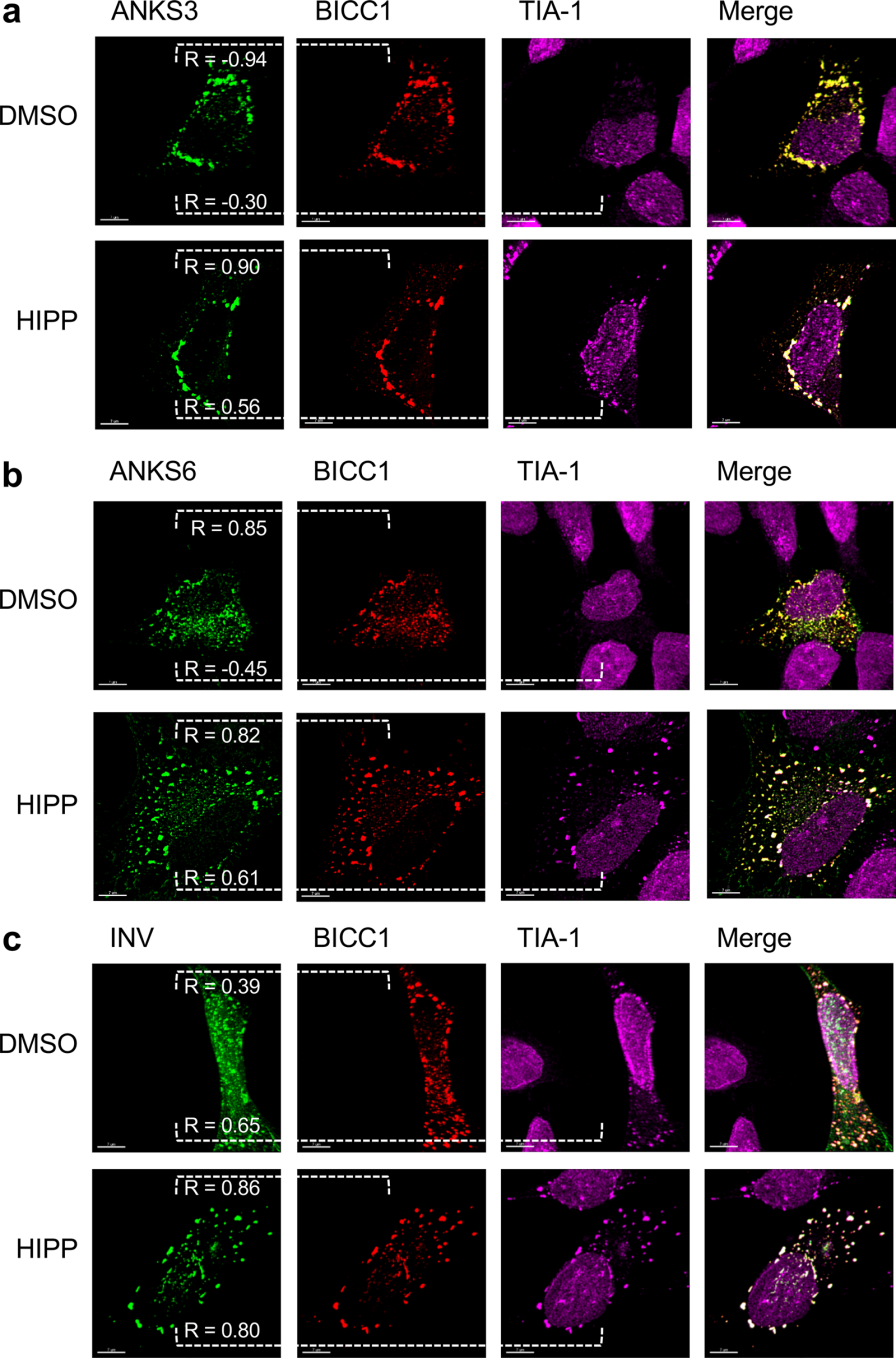
BICC1-mediated recruitment of NPH proteins into stress granules. (**a**) Co-expression of BICC1 recruited ANKS3 into granules that co-localized with TIA-1 in response to 1 mM hippuristanol. The experiment was performed two times, analyzing a total of 18 z-stacks. (**b**) Co-expression of BICC1 recruited ANKS6 into granules that co-localized with TIA-1 in response to 1 mM hippuristanol. The experiment was performed two times, analyzing a total of 17 z-stacks. (**c**) GFP-tagged INVERSIN and RFP-tagged BICC1 co-localized in granules that overlapped with TIA-1-positive stress granules even in the absence of hippuristanol. Hippuristanol treatment (1 mM for 1 h) increased the number of TIA-1-positive stress granules that largely co-localized with INVERSIN and BICC1. In this experiment, a total of 19 z-stacks were analyzed.

Co-transfection of AGO2 and BICC1 revealed that AGO2 partially accumulated in TIA-1-positive stress granules in the presence of BICC1; after exposure to hippuristanol, AGO2 and BICC1 more extensively co-localized with cytoplasmic stress granules (Fig. 6). While INVERSIN assumed a diffuse cellular localization in the presence of DMSO, it accumulated in TIA-1-positive granules after treatment with hippuristanol without co-transfection of AGO2 or BICC1 (Fig. 7a). Co-transfection of AGO2 resulted in a more predominant cytoplasmic localization of INVERSIN and largely identical distribution of both proteins in the presence of DMSO, and accumulation of both proteins in stress granules in response to hippuristanol (Fig. 7b). While ANKS3 alone assumed a largely diffuse cytoplasmic localization in the presence of either DMSO or hippuristanol (Fig. 4a), co-transfection of BICC1 caused its redistribution into granules; in the presence of hippuristanol, most of these granules became TIA-1-positive, indicating the association of ANKS3 with stress granules in response to translational inhibition in the presence of BICC1 (Fig. 8a). ANKS6 revealed a very similar pattern: co-transfection of BICC1 caused a more granular pattern. After treatment with hippuristanol, most of these granules stained positive for the stress granule marker TIA-1 (Fig. 8b). In contrast to ANKS3 and ANKS6, INVERSIN accumulated in stress granules after hippuristanol treatment in the absence of AGO2 or BICC1 (Fig. 7A). Co-transfection of BICC1 caused a partial co-localization with TIA-1-positive granules in the absence of hippuristanol that became more extensive after translational inhibition (Fig. 8c). While all four proteins appear to play a role in stress responses, BICC1 seems to recruit ANKS3 and ANKS6 to stress granules in response to translational inhibition, while INVERSIN assumed this localization in response to stress independently of BICC1. The role of endogenous ANKS6 in stress granule formation was supported by exposing murine inner medullary collecting duct (IMCD3) cells to hippuristanol after depletion of ANKS6, using a tetracycline-inducible *Anks6* knockdown IMCD3 cell line^28^. While virtually all cells displayed stress granules after hippuristanol treatment (97.5%), this percentage was reduced to 65% in cells lacking *Anks6* (Supplementary Fig. 11).

## Discussion

NPH gene products generally localize to the cilium or cilia-associated compartments, where they appear to form complex networks to gate access to the ciliary axoneme. However, several family members assume localization outside of the cilium, including cell–cell contacts and the nucleus. Family members such as SDCCAG8, NPHP9/NEK8, ZNF423 and CEP164 have been implicated in DNA damage responses^7–9^, suggesting that NPH proteins may assume different functions in developing versus mature organs, controlling embryogenesis as well as tissue maintenance.

ANKS6 (NPHP16) not only interacted with other NPH family members, but also associated with proteins involved in RNA processing. This observation led us to examine whether ANKS6 itself interacts with RNA. Since ANKS6 shares the SAM domain with the established RNA-binding protein BICC1, we compared the RNA-binding properties of ANKS6 with BICC1, while ARL13B and BBS3, lacking ankyrin repeats and any known RNA-binding domains served as negative controls. Since INVERSIN and ANKS3 share the ankyrin repeats with ANKS6, we compared the RNA-binding properties of all six proteins, using FLASH, a novel cross-linking method followed by immunoprecipitation, cDNA library construction and next-generation sequencing^24^. As expected, BICC1 bound a large number of molecules that were not found in precipitates of the negative controls. ARL13B and BBS3 displayed marginal RNA-binding properties with a very small number of interacting RNAs that likely non-specifically interacted with these two proteins. In contrast to ARL13B and BBS3, ANKS3, ANKS6 and INVERSIN bound a significant higher number of RNA molecules, displayed distinct binding motifs and interacted with RNAs that were shared with BICC1. The presence of *AGO2* mRNA in the BICC1 precipitate and the known localization of BICC1 to RNA-processing granules (P-bodies) led us to explore this interaction. Self-polymerization of the BICC1 by the SAM domain facilitates accumulation of BICC1 in cytoplasmic foci^19,29^. We confirmed that BICC1 accumulates in cytoplasmic granules. These granules co-localize with the stress granule marker TIA-1 after exposure to translational inhibition by hippuristanol. This observation suggests a dual function of BICC1 with involvement in RNA processing during cellular homeostasis as well as a function in response to cellular stress.

Stress granules (SG) are formed in response to many forms of stress to minimize energy expenditure and to facilitate cell survival^30^. SG assembly is initiated by granule nucleation. While RNA-binding proteins with prion-like domains together with untranslated mRNAs form the core, proteins with intrinsically disordered domains form the more dynamic SG shell^31^. BICC1 alone can associate with TIA-1-positive SGs, while AGO2 assumed this localization only after exposure to hippuristanol. We assume that the facultative recruitment of BICC1 into P-bodies or SGs may be related to its role in silencing RNAs^29^. Although interacting with AGO2 and weakly binding to *AGO2* mRNA, neither ANKS3 nor ANKS6 localized to stress granules even after exposure to hippuristanol. INVERSIN assumed a diffuse cytoplasmic distribution with a substantial portion of protein localizing to the nucleus. However, in contrast to ANKS3 and ANKS6, the association of INVERSIN with stress granules in response to hippuristanol did not require co-expression of BICC1.

BICC1 affects the localization of ANKS3 and ANKS6^19^. In the absence of hippuristanol, BICC1 recruited both proteins into TIA-1-negative granules, likely representing RNA-processing bodies. After exposure to hippuristanol, BICC1 recruited ANKS3 and ANKS6 to TIA-1-positive stress granules. These observations suggest that ANKS3 and ANKS6 alter their subcellular localization in response to stress in the presence of BICC1. All four proteins bound a large number of small nucleolar RNAs (snoRNAs) that are predominantly found in the nucleus, where they function as guide RNAs for methylation/pseudouridylation of ribosomal RNAs. In addition to these housekeeping functions and production of ribosomes, snoRNAs can act as precursors of microRNAs, and have been implicated in the control of cell fate and oncogenesis^32^. For example, *miR-605*, an H/ACA box snoRNA-derived miRNA can target *MDM2* mRNA to mediate stabilization of p53 in response to cellular stress^33^. BICC1, interacting with the *MDM2* mRNA, could play an additional role in p53 regulation.

Our findings reveal that similar to BICC1, the three ankyrin-repeat proteins ANKS3, ANKS6 and INVERSIN can interact with RNA molecules, and associate with stress granules in response to translational inhibitors. Our results also uncover the crucial function of BICC1 in controlling the subcellular localization of these NPH proteins in response to stress. Future work needs to delineate whether induction of cellular stress can rescue BICC1 deficiency, or trigger BICC1-dependent functions to overcome manifestations caused by defective ANKS3/ANKS6/INVERSIN functions.

## Materials and methods

### Cell lines

Stable inducible cell lines were generated using the commercial cell line Flp-In T-REx 293 according to the manufacturer’s instructions (Invitrogen), using the transgenes *mBicc1*, *hANKS3*, *hANKS6*, *hINVERSIN*, *hBBS3* and *hARL13B*. All transgenes were subcloned into the Flp-In pcDNA5/FRT/TO expression vector with a C-terminal 3xFLAG-Histidin-Biotin-Histin (HBH) tag and co-transfected with a pOG44 vector using Lipofectamine 3000 (Invitrogen). A control cell line was generated, using the empty pcDNA5/FRT/TO vector. The host cell line was maintained in Dulbecco’s Modified Eagle Medium (DMEM) supplemented with 2 mM l-glutamine, 10% (v/v) FBS (Sigma-Aldrich), 1% (v/v) penicillin/streptomycin under zeocin and blasticidin selection at 37 °C, 5% CO_2_, and 95% humidity. The hygromycin selection began 48 h after transgene transfection. Protein expression was induced with 0.1 µg/ml doxycycline for 17 h and verified by immunoblotting. Generation of the *Anks6* IMCD3 knockdown cell line was recently described^28^.

### Co-immunoprecipitation and Western blot analysis

HEK 293 T cells were cultured, transfected and lysed according to Ref.^34^. The lysates were centrifuged at 15,000*g* for 15 min at 4 °C, and the supernatants were subjected to ultracentrifugation at 100,000*g* for 30 min at 4 °C, followed by incubation with 20 µl of Flag M2 sepharose beads (Sigma-Aldrich) for 2 h at 4 °C. The beads were washed with lysis buffer; 2 × Laemmli with dithiothreitol was used to elute the bound proteins. SDS-PAGE was performed to resolve the proteins; interactions were detected by Western blot analysis.

### Immunofluorescence

HeLa cells were split onto poly-l-Lysine-coated glass slides in 6-well plates at 3.5 × 10^5^ cells per well. All cDNAs were subcloned into pcDNA6 (Invitrogen) (c-terminally RFP-tagged mBicc1, hANSK6, hANKS3, hINV, BBS3 and N-terminally GFP-tagged AGO2, rAnks6 and mAnks3). The plasmids were transfected, using FUGENE HD Transfection Kit (Promega). 24 h post transfection the cells were treated with either 1 mM hippuristanol (dissolved in DMSO)^27^, or DMSO alone for 1 h. The cells were fixed with 4% PFA for 5 min, permeabilized with 0,1% Triton-X 100 (15 min), washed with PBS and blocked with blocking solution (5% BSA, 5% normal donkey serum, 1% Gelatin from Cold Water Fish Skin (SIGMA) in PBS with Ca/Mg) for 2 h at room temperature. Staining was performed with polyclonal rabbit anti-TIA-1 antibody (1:200, ab40693, Abcam) at 4 °C overnight, followed by staining with Alexa Fluor 647-conjugated AffiniPure Fab Fragment Donkey Anti-Rabbit IgG (H + L) (1:500, Jackson Immuno Research) for 1 h at room temperature. The slides were mounted with ProLong Diamond Antifade Mountant (Molecular Probes). Confocal microscopy was done on Leica TCS SP8 gated STED with inverted Leica DMI6000 with HC PL Apo 93×/1.30 mott Corr glycerol objective. Deconvolution of z-stacks (8 slices) was carried out with Huygens Professional (SVI) manual background value 20 (remaining values: default setting) and exported as Maximum Intensity Projection with IMARIS 9.2.0 at MIAP Freiburg. To determine the extent of co-localization, the Pearson’s R value was calculated for each experiment R values < 0.30 denotes no correlation, R values between 0.30 and 0.60 indicate moderate to good correlation, R values > 0.60 indicate good to very good correlation. The Pearson correlations were calculated using the Coloc2 plugin in Fiji^35^. A region of interest excluding the nucleus was selected for each experiment.

### Immunoprecipitation of cross-linked RNA

Flag-tagged BICC1 Flp-In T-REx-293 cells were induced with 0.1 µg/ml doxycycline for 17 h. The cells were harvested in PBS and washed, then crosslinked with 0.5% formaldehyde for 10 min. The reaction was quenched by addition of 0.25 M glycine for 5 min, followed by washing with ice-cold PBS. The cells were lysed in 1.5 ml low stringency RIPA buffer (50 mM Tris–HCl, pH 7.5, 150 mM NaCl, 1% IGEPAL CA-630, 0.5% sodium deoxycholate, 0.05% SDS, 1 mM EDTA, pH 8.0) and protease inhibitors (cOmplete, Roche Diagnostics). The cells were homogenized by sonication in two cycles with pulse time of 0.5 s “on” and 0.5 s “off” for 15 s at 50% amplitude with a 2 min incubation on ice in between. Lysates were centrifuged for 15 min at 20,000*g*, 4 °C. For immunoprecipitation 50 µl Dynabeads Protein G (Invitrogen) were incubated with 5 µg rabbit α-FLAG pAb (Sigma) in PBS at 4 °C for 3 h, washed twice and then treated with 0.5 µl SUPERaseIn RNase Inhibitor (20 U/μl, Invitrogen) for 10 min. A negative control as stated above, but without addition of antibody. For pre-clearing 30 µl uncoated Dynabeads Protein G (Invitrogen) were treated with RNase Inhibitor (20 U/μl, Invitrogen) for 10 min. The lysates were mixed with the pre-clearing beads and 100 µg/ml yeast tRNA for 1 h at 4 °C. 10% of the lysates were used for input control. The pre-cleared lysates were added to either the antibody-bound beads or the control beads, and incubated for 1 h at 4 °C. The samples were spun down 15 min at 4000*g* and 4 °C, and washed three times using high stringency RIPA buffer (50 mM Tris–HCl, pH 7.5, 1% IGEPAL CA-630, 1% sodium deoxycholate, 0.1% SDS, 1 mM EDTA, pH 8.0, 1 M NaCl, 1.5 M urea, protease inhibitors) followed by one wash with PBS. The beads were resuspended in elution buffer (50 mM Tris–HCl, pH 7.5, 5 mM EDTA, 10 mM dithiothreitol, 1% SDS), and incubated at 70 °C for 45 min. RNA was extracted, using TRIzol Reagent (Invitrogen), followed by an in-column DNAse I treatment and enrichment, using RNA Clean & Concentrator-25 (Zymo Research). Reverse transcription with hexamer primers was performed with the SuperScript III First-Strand Synthesis System (Invitrogen). A control reaction omitting the enzyme was executed to confirm the absence of contaminating genomic DNA. Five µg of cDNA was used to amplify the specific AGO2 transcripts identified in FLASH via PCR using the forward primer 5′-CGAAAATCACCCACCCCAC-3′ and reverse primer 5′-AGCTGGTAGGTTAGGATCTGC-3′ and then separated in a 3% agarose gel.

### FLASH assays

FLASH experiments were performed as described^36^. Doxycycline-induced HEK 293 Flp-In T-REx cell lines, were rinsed with PBS and cross-linked with 200 mJ/cm^2^ UV-C irradiation. The cells were pelleted and resuspended in lysis buffer (PBS, 0.3 M NaCl, 1% Triton-X, 0.1% Tween-20) and protease inhibitors (cOmplete, Roche Diagnostics), and homogenized by 5 cycles of sonication with a pulse time of 30 s “on” and 30 s “off” in a water bath sonicator at 4 °C. The samples were then first precipitated with His-Tag Isolation and Pulldown Dynabeads (ThermoFisher) for 10 min on ice, and subsequently washed with lysis buffer for 4 min at 4 °C. The bound proteins were eluted with 250 mM imidazole in lysis buffer for 5 min on ice. The eluate was incubated with Streptavidin C1 Dynabeads (ThermoFisher) for 45 min at 4 °C under rotation, followed by two washing steps with high salt buffer (HSB, 50 mM Tris–HCl, pH 7.5, 1 M NaCl, 1% Triton-X 100, 0.1% SDS, 0.5% sodium deoxycholate, 1 mM EDTA) and non-denaturing buffer (NDB, 50 mM Tris–HCl, pH 7.5, 100 mM NaCl, 0.1% Tween-20), respectively. The cross-linked RNA was briefly digested with RNase-I and washed again with high salt buffer and NDB. The 3′ ends of the RNA were repaired with T4 poly-nucleotide kinase for 20 min at 37 °C, centrifuged at 1100 rpm, followed by washing steps with HSB and NDB. The FLASH adapters described in Ref.^36^ were ligated to the cross-linked RNA using T4 RNA Ligase I for 1 h at 25 °C. To remove excess adapters, the experimental samples and negative controls were merged by resuspension in HSB on ice and subsequently washed twice with LDS buffer (20 mM Tris–HCl, pH 7.5, 0.5 M LiCl, 0.5% LiDS, 1 mM EDTA), followed by washing with HSB and NDB, respectively. The merged sample was treated with rSAP (New England Biolabs) for 30 min at 30 °C. The RNA was released by Proteinase K digestion and purified with the Oligo Clean & Concentrator kit (Zymo), following by reverse transcription using SuperScript III First-Strand Synthesis System (Invitrogen) and digested with RNase H. The cDNA was purified with the Oligo Clean & Concentrator kit (Zymo), and circularized with CircLigase (Biozym/epicentre) for 16 h. The circularized cDNA was PCR-amplified, purified with Agencourt AMPure XP beads (Beckman Coulter), followed by quality control using a Bioanalyzer and sequenced on an Illumina NextSeq 500 Analyzer in paired-end mode. The FLASH experiments analysis was adapted from Ref.^36^, and described in Supplementary Fig. 12. At least eight individual FLASH experiments were performed for BICC1, INV; ANKS6, ANKS3; six experiments were performed for BBS3 and ARL13B.

### Zebrafish experiments

The transgenic zebrafish line *wt1b::GFP* was maintained as described^17^. Fertilized eggs were micro-injected at the one-cell stage with 4 nl of injection solution containing morpholino oligonucleotides (MO; Gene Tools, Philomath, USA) diluted in 200 mmol/l KCl, 0.1% Phenol Red, and 10 mmol/l HEPES (4-(2-hydroxyethyl)-1-piperazineethanesulfonic acid).The *anks3* antisense morpholino oligonucleotide (*anks3 SBM*; 5′-GCTCAGACATCCTCCTCTGGAAATC-3′) and the standard control MO (5′-CCTCTTACCTCAGTTACAATTTATA-3′) were previously described^17^. The sequence of the *ago2* splice blocking MO (*ago2 SBM*) is 5′-AGGTTGAGTCAAAAGATTTTACCGG-3′. To reduce the unspecific effects of the reagents, 0.4 pmol of zebrafish *p53* MO (5′-GCGCCATTGCTTTGCAAGAATTG-3′, Gene Tools) were added to the injection solution. All zebrafish experiments were approved by the regional council in Freiburg and were performed according to approved guidelines.

### Mass spectrometry analyses

Samples were separated on 4–12% gradient gels, gel lanes were cut into 10 equal slices, the proteins were in-gel digested with trypsin and the resulting peptide mixtures were processed on STAGE tips and analyzed by LC–MS/MS^37^. Mass spectrometry (MS) analyses were performed on an LTQ Orbitrap XL mass spectrometer coupled to an Agilent 1200 nanoflow-HPLC. The mass spectrometer was operated in the data-dependent mode, switching automatically between MS (max. of 1 × 10^6^ ions) and maximally five MS/MS (target value 5000). MS raw data were uploaded into the MaxQuant software version 1.4.1.2 for peak detection, generation of peak lists of mass error corrected peptides, and for database searches^38^. A full-length UniProt mouse database additionally containing common contaminants such as keratins and enzymes used for in-gel digestion (based on UniProt mouse version Dec. 2014) was used as reference. Triple SILAC was chosen as quantitation mode. Three missed cleavages were allowed, enzyme specificity was trypsin/P. Peptide and protein false discovery rates, based on a forward-reverse database, were set to 0.01, minimum peptide length was set to 7, minimum number of peptides for identification and quantitation of proteins was set to one which must be unique, minimum ratio count was set to one, and identified proteins were re-quantified.

### Consent for publication

Consent from all authors were obtained.

## Supplementary information

Supplementary Legends.

Supplementary Figures.

Supplementary Table 1.

Supplementary Table 2.

## References

[1] Wolf MT (2015). Nephronophthisis and related syndromes. Curr Opin Pediatr.

[2] Scheidel N, Blacque OE (2018). Intraflagellar transport complex A genes differentially regulate cilium formation and transition zone gating. Curr. Biol..

[3] Braun DA, Hildebrandt F (2017). Ciliopathies. Cold Spring Harb. Perspect. Biol..

[4] Simms RJ, Eley L, Sayer JA (2009). Nephronophthisis. Eur. J. Hum. Genet..

[5] Schermer B (2005). Phosphorylation by casein kinase 2 induces PACS-1 binding of nephrocystin and targeting to cilia. EMBO J..

[6] Nurnberger J, Bacallao RL, Phillips CL (2002). Inversin forms a complex with catenins and N-cadherin in polarized epithelial cells. Mol. Biol. Cell.

[7] Chaki M (2012). Exome capture reveals ZNF423 and CEP164 mutations, linking renal ciliopathies to DNA damage response signaling. Cell.

[8] Choi HJ (2013). NEK8 links the ATR-regulated replication stress response and S phase CDK activity to renal ciliopathies. Mol. Cell.

[9] Airik R (2014). Renal-retinal ciliopathy gene Sdccag8 regulates DNA damage response signaling. J. Am. Soc. Nephrol..

[10] Walz G (2017). Role of primary cilia in non-dividing and post-mitotic cells. Cell Tissue Res..

[11] Blacque OE, Sanders AA (2014). Compartments within a compartment: What *C. elegans* can tell us about ciliary subdomain composition, biogenesis, function, and disease. Organogenesis.

[12] Tsuji T, Matsuo K, Nakahari T, Marunaka Y, Yokoyama T (2016). Structural basis of the Inv compartment and ciliary abnormalities in Inv/nphp2 mutant mice. Cytoskeleton (Hoboken).

[13] Simons M (2005). Inversin, the gene product mutated in nephronophthisis type II, functions as a molecular switch between Wnt signaling pathways. Nat. Genet..

[14] Lienkamp S (2010). Inversin relays Frizzled-8 signals to promote proximal pronephros development. Proc. Natl. Acad. Sci. U. S. A..

[15] Hoff S (2013). ANKS6 is a central component of a nephronophthisis module linking NEK8 to INVS and NPHP3. Nat. Genet..

[16] Ramachandran H (2015). Anks3 alters the sub-cellular localization of the Nek7 kinase. Biochem. Biophys. Res. Commun..

[17] Yakulov TA (2015). Anks3 interacts with nephronophthisis proteins and is required for normal renal development. Kidney Int..

[18] Shamseldin HE, Yakulov TA, Hashem A, Walz G, Alkuraya FS (2016). ANKS3 is mutated in a family with autosomal recessive laterality defect. Hum. Genet..

[19] Rothe B (2018). Crystal structure of Bicc1 SAM polymer and mapping of interactions between the ciliopathy-associated proteins Bicc1, ANKS3, and ANKS6. Structure.

[20] Saffman EE (1998). Premature translation of oskar in oocytes lacking the RNA-binding protein bicaudal-C. Mol. Cell. Biol..

[21] Maisonneuve C (2009). Bicaudal C, a novel regulator of Dvl signaling abutting RNA-processing bodies, controls cilia orientation and leftward flow. Development.

[22] Chicoine J (2007). Bicaudal-C recruits CCR4-NOT deadenylase to target mRNAs and regulates oogenesis, cytoskeletal organization, and its own expression. Dev. Cell.

[23] Aviv T (2003). The RNA-binding SAM domain of Smaug defines a new family of post-transcriptional regulators. Nat. Struct. Biol..

[24] Aktas T (2017). DHX9 suppresses RNA processing defects originating from the Alu invasion of the human genome. Nature.

[25] Machanick P, Bailey TL (2011). MEME-ChIP: Motif analysis of large DNA datasets. Bioinformatics.

[26] Leung AK, Calabrese JM, Sharp PA (2006). Quantitative analysis of Argonaute protein reveals microRNA-dependent localization to stress granules. Proc. Natl. Acad. Sci. U. S. A..

[27] Bordeleau ME (2006). Functional characterization of IRESes by an inhibitor of the RNA helicase eIF4A. Nat. Chem. Biol..

[28] Schlimpert M (2019). Metabolic perturbations caused by depletion of nephronophthisis factor Anks6 in mIMCD3 cells. Metabolomics.

[29] Rothe B (2015). Bicc1 polymerization regulates the localization and silencing of bound mRNA. Mol. Cell Biol..

[30] Mahboubi H, Stochaj U (1863). Cytoplasmic stress granules: Dynamic modulators of cell signaling and disease. Biochim. Biophys. Acta Mol. Basis Dis..

[31] Gilks N (2004). Stress granule assembly is mediated by prion-like aggregation of TIA-1. Mol. Biol. Cell.

[32] Williams GT, Farzaneh F (2012). Are snoRNAs and snoRNA host genes new players in cancer?. Nat. Rev. Cancer.

[33] Xiao J, Lin H, Luo X, Luo X, Wang Z (2011). miR-605 joins p53 network to form a p53:miR-605:Mdm2 positive feedback loop in response to stress. EMBO J..

[34] Yasunaga T (2015). The polarity protein Inturned links NPHP4 to Daam1 to control the subapical actin network in multiciliated cells. J. Cell Biol..

[35] Schindelin J (2012). Fiji: an open-source platform for biological-image analysis. Nat. Methods.

[36] Ilik IA, Aktas T, Maticzka D, Backofen R, Akhtar A (2019). FLASH: Ultra-fast protocol to identify RNA–protein interactions in cells. Nucleic Acids Res..

[37] Thriene K (2018). Combinatorial omics analysis reveals perturbed lysosomal homeostasis in collagen VII-deficient keratinocytes. Mol. Cell Proteom..

[38] Cox J, Mann M (2008). MaxQuant enables high peptide identification rates, individualized p.p.b.-range mass accuracies and proteome-wide protein quantification. Nat. Biotechnol..

