# Supplementary material for: Nephronophthisis gene products display RNA-binding properties and are recruited to stress granules
Source: Sci Rep. 2020 Sep 29;10:15954. doi: 10.1038/s41598-020-72905-8 (PMC7524721; doi:10.1038/s41598-020-72905-8)
Supplement: Supplementary file 1 — Supplementary Legends. [file 41598_2020_72905_MOESM1_ESM.docx]

**Supplemental Figure Legends**

**Supplemental Figure 1.** Domain architecture of BICC1, INVERSIN (INV), ANKS3 and ANKS6. While only BICC1 contains RNA-binding K homology (KH) domains, it shares the SAM domain with ANKS3 and ANKS6. Both BICC1 and ANKS6 contain several regions of low complexity (magenta), while INV, ANKS3 and ANKS6 share several ankyrin repeats (ANK). Images were generated using SMART (www.smart.embl.de).

**Supplemental Figure 2.** SILAC-based mass spectrometry of endogenous ANKS6 precipitated from IMCD cells. (**a**) Endogenous ANKS6 was precipitated from IMCD cells incubated in SILAC media. The uncropped Western blots are shown in Suppl. Fig. 13. (**b**) GO-term analysis of putative ANKS6 interacting proteins. SILAC-based mass spectrometry of endogenous ANKS6 precipitated from IMCD cells supports the results of the ANKS6 interactome in kidney lysates. Molecular functions involved in RNA binding and processing ranged among the top terms. Analysis was performed using STRING (www.string-db.org/).

**Supplemental Figure 3.** RNA-binding properties of Flag-tagged proteins by FLASH. **(a)** Flp-in cell lines stably expressing 3xFlag-HBH-tagged proteins. For the FLASH assay, proteins were twice precipitated, using cobalt and streptavidin beads. For Western blot analysis, beads were resuspended in Laemmli buffer; proteins were detected, using an anti-Flag monoclonal antibody. Uncropped Western blots are shown in Suppl. Fig. 13. (**b**) Pearson correlation heatmap. Heatmap of Pearson correlation for de-duplicated sample files of the FLASH experiments was plotted using deep-tools on the Galaxy platform. Blue depicts a strong, yellow a moderate, and red no correlation.

**Supplemental Figure 4.** RNA Binding motifs for BICC1, INVERSIN, ANKS3 and ANKS6. Depicted are the top binding motifs ranked 3-10 according to the RNA Centric Analysis System (RCAS) report tool on Galaxy.

**Supplemental Figure 5.** RNA-binding characteristics of BICC1, INVERSIN, ANKS3 and ANKS6. (**a**) Depicted is the depth of coverage of query regions at and around transcription start and end sites (RNA Centric Analysis System (RCAS) report tool on Galaxy, BIMSB, Max-Delbrück Center for Molecular Medicine, Berlin). (**b**) Depicted are query regions overlaid with the genomic coordinates of features. Features ≥ 100 bp were divided into 100 bins of equal length, and the mean coverage score for each bin was represented by ribbons; the thickness of the ribbon indicates the 95% confidence interval. The coverage profile was plotted in the 5’ to 3’ direction (RNA Centric Analysis System (RCAS) report tool on Galaxy, BIMSB, Max-Delbrück Center for Molecular Medicine, Berlin).

**Supplemental Figure 6.** Heatmap based on 70 transcripts shared between BICC1, ANKS6, INV, and ANKS3. The heatmap was generated using the top genes that overlap query regions (RNA Centric Analysis System (RCAS) report tool on Galaxy, BIMSB, Max-Delbrück Center for Molecular Medicine, Berlin) and are shared between BICC1, ANKS6, INV and ANKS3 and absent in the negative controls ARL13B and BBS3. The heatmap suggests that the RNA binding properties of INV and ANKS3 are more closely related to each other than to BICC1 and ANKS6 (www.heatmapper.ca).

**Supplemental Figure 7. Analysis of interacting RNA molecules.** (**a**) Top 100 GO terms for RNA molecules interacting with BICC1, INVERSIN, ANKS3 and ANKS6. (**b**) Flag-tagged proteins were precipitated, and interacting AGO2 mRNA was reverse-transcribed and amplified by PCR, yielding a 145 bp band. Controls without reverse transcriptase (RT) were negative. There was a faint signal in the ANKS6 precipitate in the absence of Flag antibody, indicating some non-specific binding to the beads; however, the signal for ANKS6 in the presence of RT and antibody was substantially stronger, suggesting binding of *AGO2* mRNA to ANKS6. Marginal binding of AGO2 mRNA was observed for ANKS3, while no binding was found for INVERSIN. All RNA-immunoprecipitations were performed three times. The uncropped electrophoresis gels are displayed in Suppl. Fig. 13. Alpha Imager V.4.1.0.2 was used for visualization.

**Supplemental Figure 8.** AGO2 and CAPRIN1 interactions. (**a**) V5-tagged AGO2 was co-transfected with Flag-tagged ANKS3, ANKS6, INVERSIN and BICC1 in HEK 293T cells. Immunoprecipitation of the four proteins immobilized AGO2. (**b**) GFP-tagged CAPRIN1 was co-transfected with Flag-tagged ANKS3, ANKS6, INVERSIN and BICC1 in HEK 293T cells. Immunoprecipitation of the four proteins immobilized CAPRIN1. The uncropped Western Blots are shown in Suppl. Fig. 13.

**Supplemental Figure 9.** Depletion of zebrafish *ago2* augments the ciliopathy phenotype of *anks3*-depleted zebrafish embryos. (**a**) Zebrafish embryo injected with 0.4 pmol of control morpholino oligonucleotide (MO) (*control*), or (**b**) *ago2* splice-blocking morpholino oligonucleotide (SBM) develop normally. (**c**) While zebrafish embryos micro-injected with higher concentrations of control MO revealed no abnormalities, (**d**) 1.2 pmol *ago2* SBM caused hydrocephalus (arrow), and curly-up tails (arrowhead). (**e**) Normal zebrafish pronephros development after micro-injection of control MO. (**f**) Proximal bilateral pronephros cysts (arrows) after combined depletion of zebrafish *ago2* and *anks3* (*ago2*/*anks3* SBM). The *wt1b:GFP* zebrafish line was used to visualize cyst formation by fluorescence microscopy. (**g**) Depletion of ago2 caused cyst formation in a concentration dependent manner. (**h**) The combination of both MOs increased the frequency of cyst formation to 35%, suggesting a synergistic genetic interaction between zebrafish *anks3* and *ago2*. Significance was calculated using Student’s t-test; n is the total number of embryos that were analyzed.

**Supplemental Figure 10.** Subcellular localization of BICC1 and AGO2 in HeLa cells. (**a**) RFP-tagged BICC1 was transfected into HeLa cells, and exposed to either DMSO or 1 mM hippuristanol for one hour. BICC1 formed small, predominantly cytoplasmic granules that did not overlap with endogenous TIA-1. In some cells BICC1 formed larger aggregates that co-localized with TIA-1, which could result from high levels of BICC1 expression. In cells treated with hippuristanol, almost all granules co-localized with TIA-1. This experiment was performed four times, analyzing a total of 58 images and 15 z-stacks. (**b**) In the presence of DMSO, AGO2 assumed a diffuse cytoplasmic localization, while endogenous TIA-1 was found almost exclusively in the nucleus. After treatment with 1 mM hippuristanol for one hour, both proteins were found in cytoplasmic stress granules. This experiment was performed four times, analyzing a total of 68 images and 24 z-stacks.

**Supplemental Figure 11.** Defective stress granule formation in *Anks6*-depleted IMCD3 cells. (**a**) IMCD3 cells with a tetracycline-inducible knockdown of *Anks6* ^1^ were treated with DMSO (Control), or hippuristanol in the presence or absence of tetracycline. After treatment with DMSO or 1 mM hippuristanol for one hour, cells were stained with a polyclonal anti-TIA-1 antibody to detect stress granule formation. Cells with or without stress granules from two independent experiments were then scored (right side of the panel). (**b**) Almost no stress granules were observed in control or *Anks6*-depleted cells. While addition of hippuristanol induced stress granule in virtually all cells (97.5%), *Anks6* depletion reduced stress granule formation to 65%. Two hundred cells were scored for each condition (Chi square test, p < 0.001). (**c**) Knockdown of *Anks6* in IMCD3 cells after exposure to tetracycline ^1^. The uncropped Western blots is shown in Suppl. Fig. 13.

**Supplemental Figure 12.** Schematic FLASH analysis workflow. The Galaxy platform ^2^ was used to perform the FLASH analysis adapted from ^3^. The adapters were trimmed by Flexbar (v2.5), and the libraries were de-multiplexed with bctools (https://github.com/dmaticzka/bctools, v0.2.0) and Flexbar (v2.5). Potential read-throughs into the barcoded regions were removed by clipping 13 nt from the 3’ ends of the first mate reads. The reads were then mapped to the reference genome hg19 using bowtie2 (v2.2.6) with the parameters: –very-sensitive –end-to-end –no-mixed –no-discordant –maxins 500. The reads for which bowtie2 could identify multiple distinct alignments as indicated by the XS:i flag were excluded. Removal of the PCR duplicates was conducted using bctools based on the random tags. The alignments of the remaining uniquely mapped reads were used to determine crosslinking events as described ^4^. PEAKachu (Galaxy tool ID: toolshed.g2.bx.psu.edu/repos/rnateam/peakachu/ peakachu/0.1.0.2, galaxy version 0.1.0.2; parameters: –no pairwise_replicates -m 2.0 -n manual –size_factors 1 1 0.75 0.75) was used to call the FLASH peaks on the alignments of crosslinking events ^5^. Replicates of the respective pull-down condition were used as foreground, and the replicates of the corresponding negative control pull-down condition were used as background. The Galaxy interface was also used to run the RNA centric annotation system (RCAS), which generates a report containing an in-depth annotation summary, sequence motif discovery as well as GO term and gen set enrichment analysis ^6^.

**Supplemental Figure 13.** Uncropped gels and Western blots. (**a**) The figure shows the uncropped electrophoresis gel that was presented in Fig. 3a. The red box indicates the cropped area. The interacting AGO2 mRNA reverse transcribed and detected in the BICC1 RNA immunoprecipitation. (**b**) Uncropped Western blot of samples used for mass spectrometry in Suppl. Fig. 2a. (**c**) Uncropped Western blots shown in Suppl. Fig. 3a. (**d**) Uncropped electrophoresis gels from RT-PCR. The red boxes specify the area that was cropped and shown in Suppl. Fig 7b. The interacting AGO2 mRNA was amplified after reverse transcription, yielding a 145 bp band. (**e**) The Western blots presented in Suppl. Fig 8a are indicated by the red boxes. The Western blots were detected with Super RX-N film (Fujifilm) and developed on the CURIX 60 (AGFA) machine. (**f**) The red boxes delineate the Western blots shown in Suppl. Fig 8b. The Western blots were detected and developed as mentioned above. (**g**) Uncropped Western blots, demonstrating the knockdown of *Anks6* in Suppl. Fig. 11b.

**Supplemental Tables**

**Supplemental Table 1.** SILAC-based mass spectrometry analysis of ANKS6-interacting proteins.

**Supplemental Table 2.** BICC1-, ANKS3-, ANKS6-, INVERSIN-, ARL13B- and BBS3-interacting RNA molecules.

**Supplemental Literature**

1 Schlimpert, M. *et al.* Metabolic perturbations caused by depletion of nephronophthisis factor Anks6 in mIMCD3 cells. *Metabolomics* **15**, 71, doi:10.1007/s11306-019-1535-0 (2019).

2 Afgan, E. *et al.* The Galaxy platform for accessible, reproducible and collaborative biomedical analyses: 2016 update. *Nucleic Acids Res* **44**, W3-W10, doi:10.1093/nar/gkw343 (2016).

3 Ilik, I. A., Aktas, T., Maticzka, D., Backofen, R. & Akhtar, A. FLASH: ultra-fast protocol to identify RNA-protein interactions in cells. *Nucleic Acids Res*, doi:10.1093/nar/gkz1141 (2019).

4 Ilik, I. A. *et al.* Tandem stem-loops in roX RNAs act together to mediate X chromosome dosage compensation in Drosophila. *Mol Cell* **51**, 156-173, doi:10.1016/j.molcel.2013.07.001 (2013).

5 Holmqvist, E. *et al.* Global RNA recognition patterns of post-transcriptional regulators Hfq and CsrA revealed by UV crosslinking in vivo. *EMBO J* **35**, 991-1011, doi:10.15252/embj.201593360 (2016).

6 Uyar, B. *et al.* RCAS: an RNA centric annotation system for transcriptome-wide regions of interest. *Nucleic Acids Res* **45**, e91, doi:10.1093/nar/gkx120 (2017).
