# Supplementary material for: Nephronophthisis gene products display RNA-binding properties and are recruited to stress granules
Source: Sci Rep. 2020 Sep 29;10:15954. doi: 10.1038/s41598-020-72905-8 (PMC7524721; doi:10.1038/s41598-020-72905-8)

Supplemental Figures

Nephronophthisis gene products display RNA-binding properties and are recruited to stress granules

Luisa Estrada Mallarino[1,2], Christina Engel[1], İbrahim Avşar Ilık[3†], Daniel Maticzka[4], Florian Heyl[4], Barbara Müller[1], Toma A. Yakulov[1], Jörn Dengjel[5+], Rolf Backofen[4], Asifa Akhtar[3,6], Gerd Walz[1,7]*

[1]Renal Division, University Freiburg Medical Center, Faculty of Medicine, University of Freiburg, Germany
[2]Faculty of Biology, University of Freiburg
[3]Max Planck Institute of Immunobiology and Epigenetics, Freiburg, Germany
[4]Institute for Informatics, Albert-Ludwigs-University, Freiburg, Germany
[5]Department of Dermatology, Medical Center, and Freiburg Institute for Advanced Studies, University of Freiburg, Germany
[6]CIBSS - Centre for Integrative Biological Signalling Studies, University of Freiburg
[7]Signalling Research Centres BIOSS and CIBSS, University of Freiburg

Supplemental Figure 1

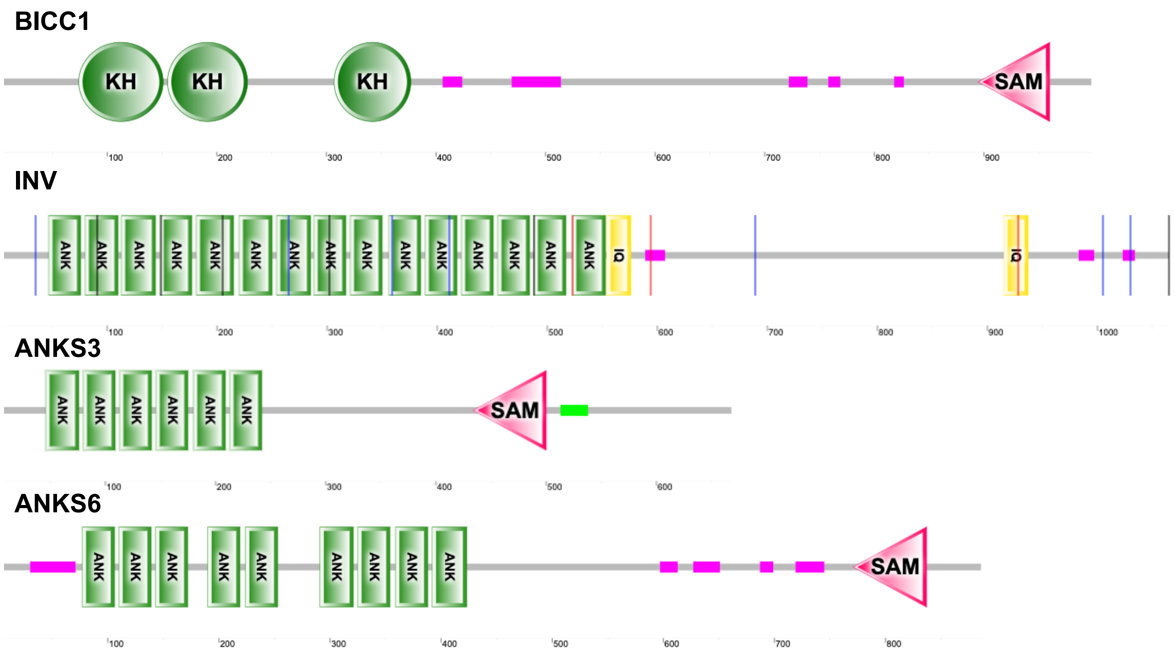

Supplemental Figure 2

a

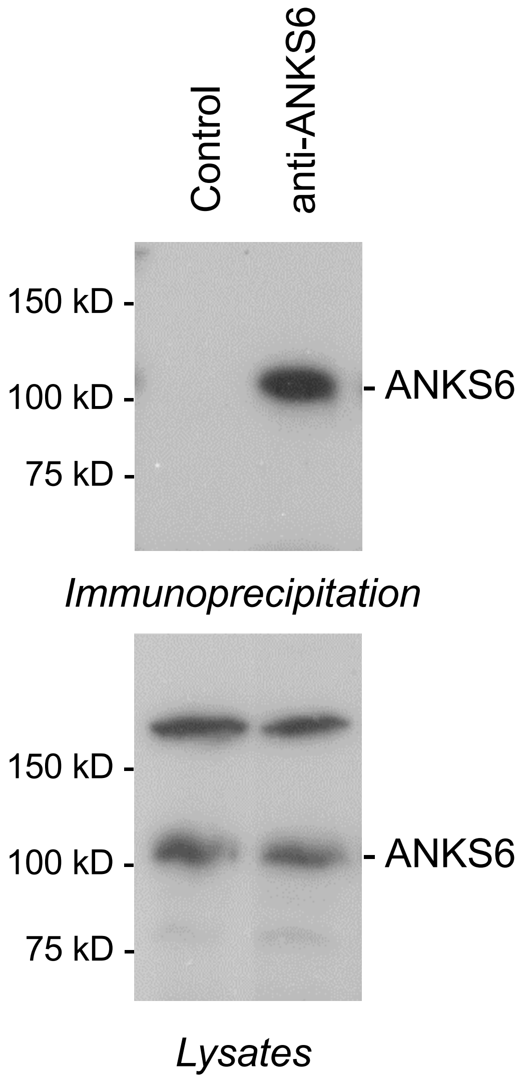

b

GO-Term	Term Description	Observed Gene Count	Background Gene Count	FDR
**Biological Process**				
GO:0043604	amide biosynthetic process	66	414	1.01e-60
GO:0006412	translation	61	313	1.43e-60
GO:0006518	peptide metabolic process	63	440	1.45e-55
GO:0043603	cellular amide metabolic process	68	644	1.46e-52
GO:1901566	organonitrogen compound biosynthetic process	73	1122	2.74e-43
**Molecular Function**				
GO:0003735	structural constituent of ribosome	58	153	3.78e-72
GO:0005198	structural molecule activity	70	546	5.04e-60
GO:0003723	RNA binding	66	986	1.40e-39
GO:1901363	heterocyclic compound binding	106	4748	3.66e-26
GO:0097159	organic cyclic compound binding	106	4818	1.03e-25
**Cellular Component**				
GO:0022626	cytosolic ribosome	58	107	1.69e-79
GO:0044391	ribosomal subunit	60	181	2.82e-72
GO:0005840	ribosome	61	215	3.04e-70
GO:0044445	cytosolic part	59	228	7.80e-66
GO:1990904	ribonucleoprotein complex	78	765	2.34e-61
**KEGG**				
mmu03010	Ribosome	55	128	3.76e-71
mmu01200	Carbon metabolism	8	118	0.00052
mmu05322	Systemic lupus erythematosus	7	92	0.00064
mmu00020	Citrate cycle (TCA cycle)	4	32	0.0063
mmu00010	Glycolysis / Gluconeogenesis	5	65	0.0065

Supplemental Figure 3

a

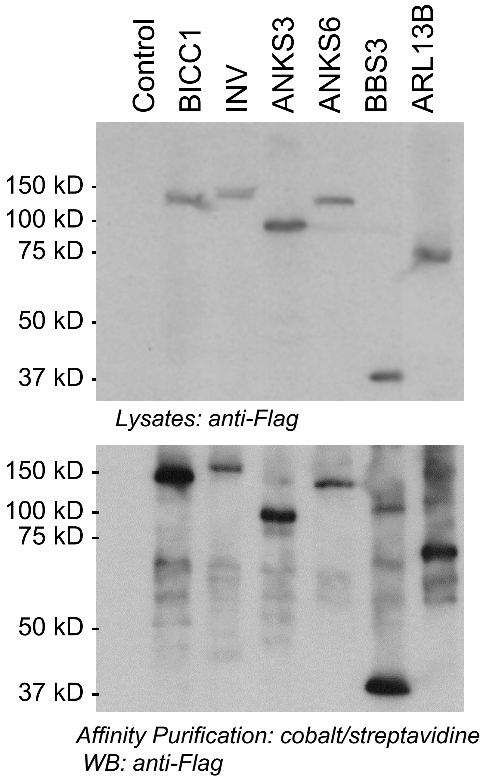

b

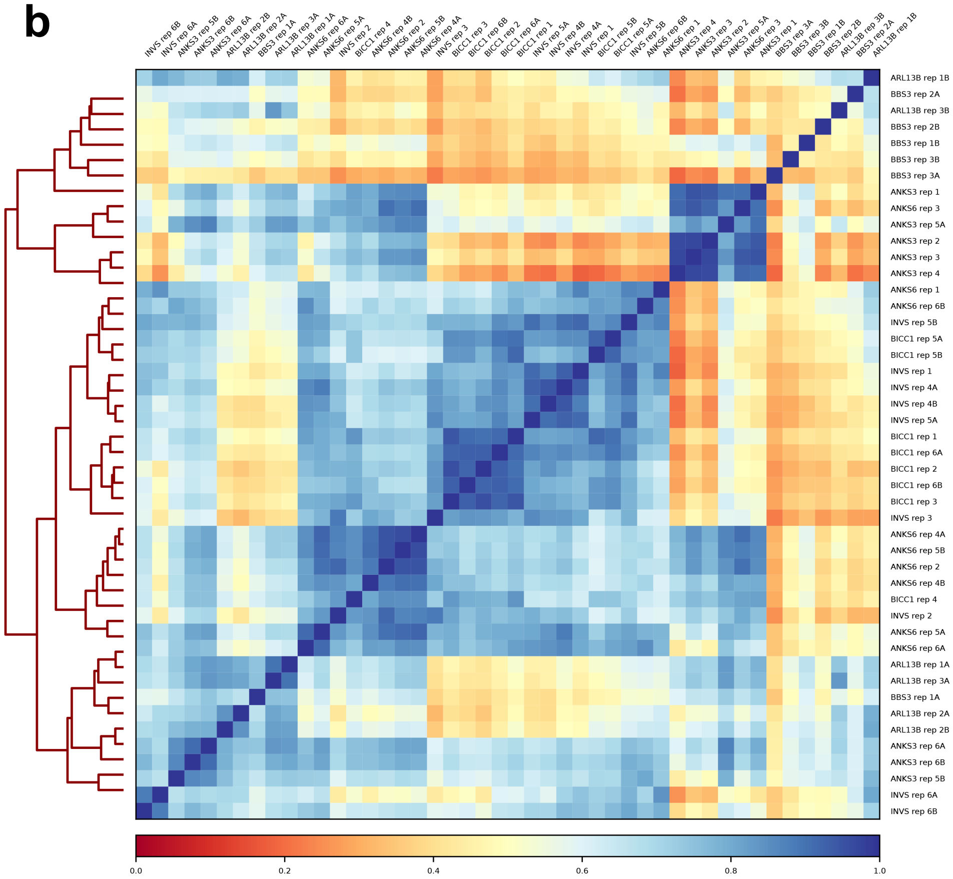

Supplemental Figure 4

BICC1

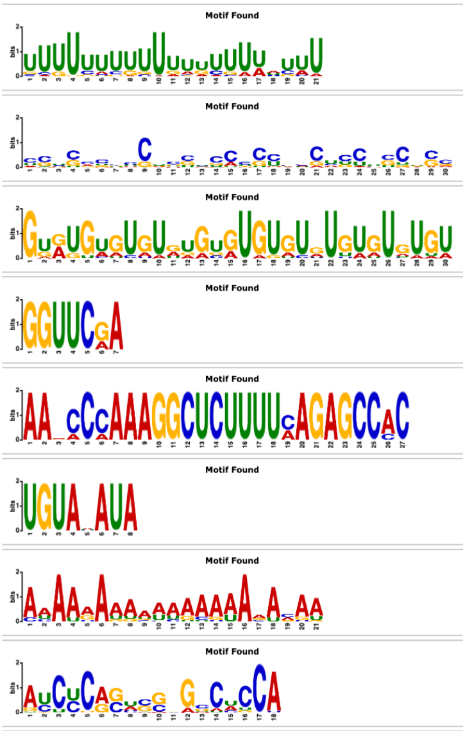

INVERSIN

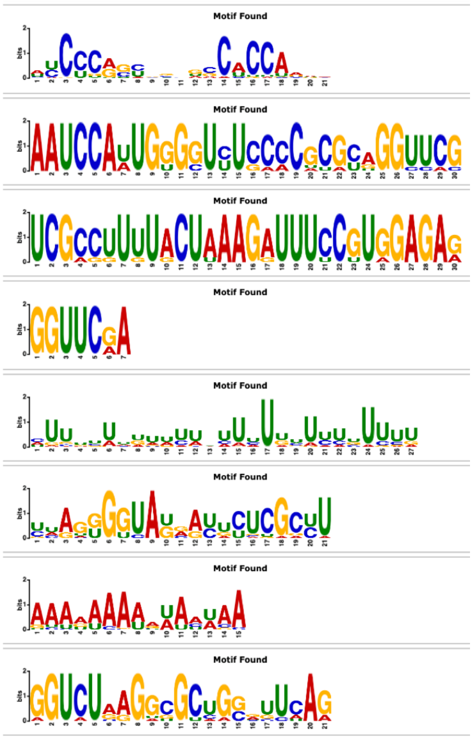

ANKS3

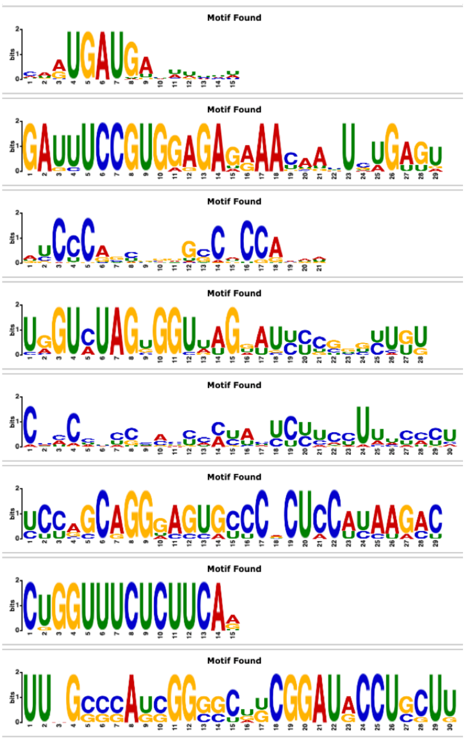

ANKS6

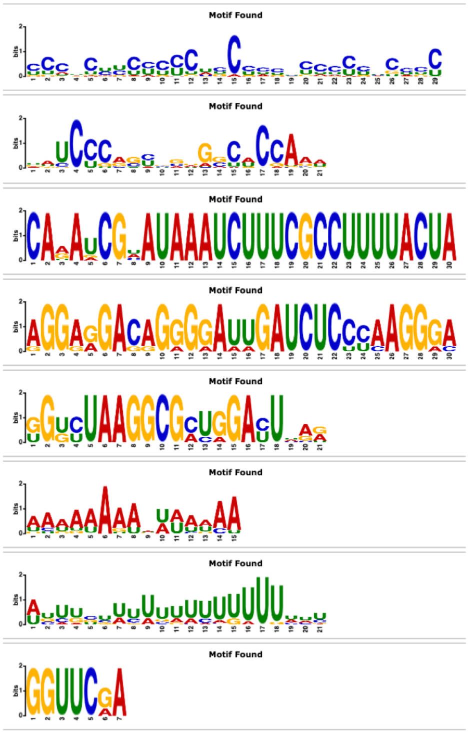

Supplemental Figure 5

BICC1

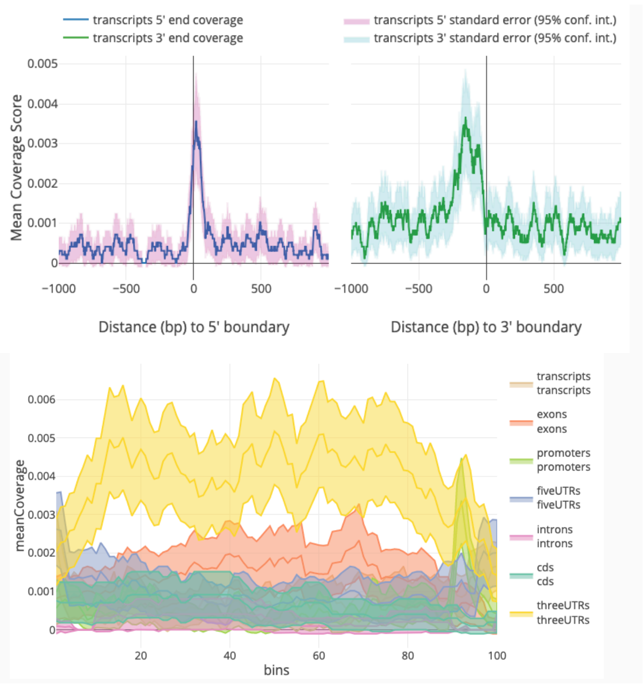

INVERSIN

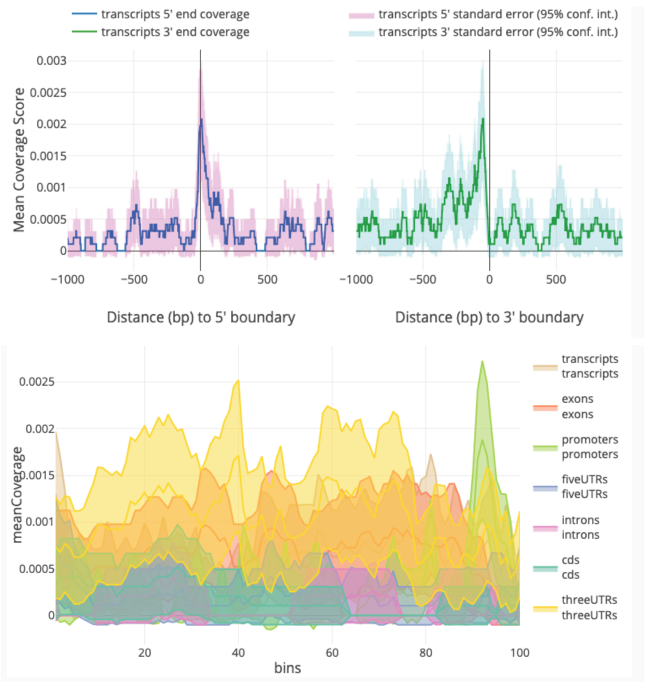

ANKS3

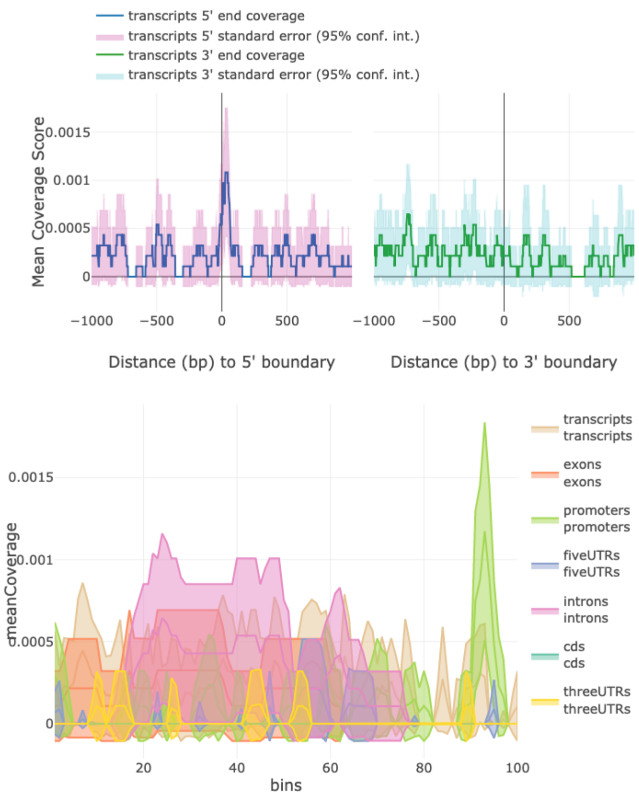

ANKS6

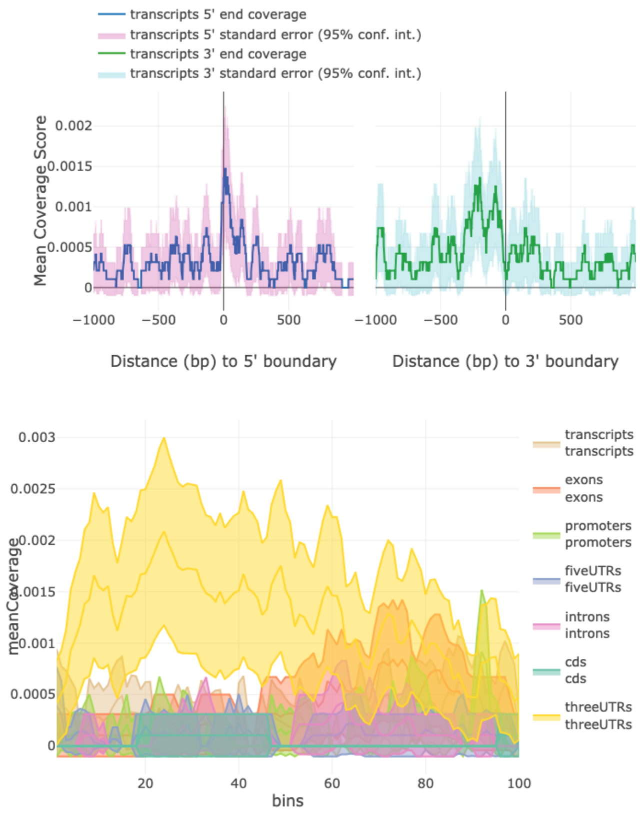

Supplemental Figure 6

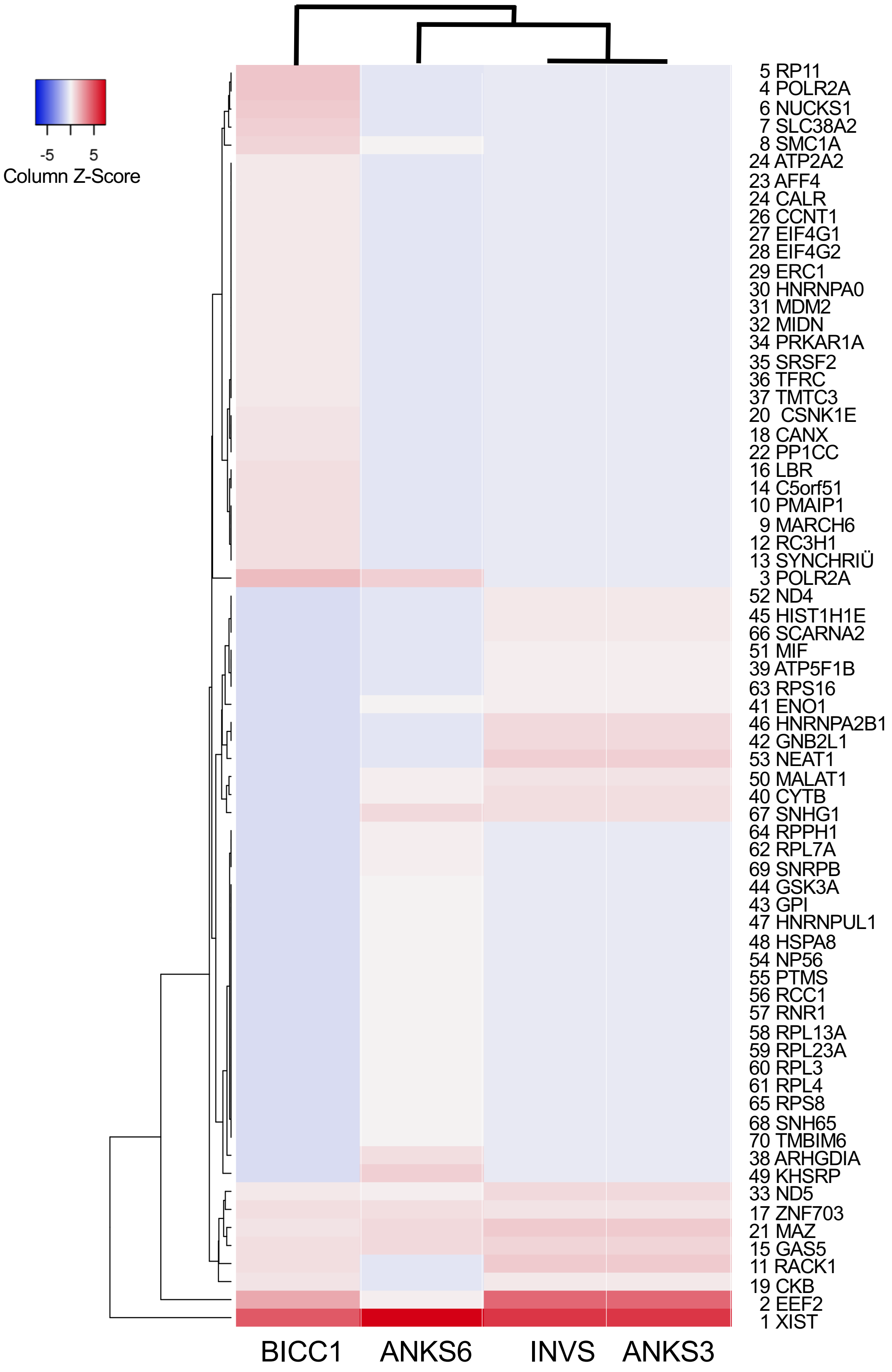

Supplemental Figure 7

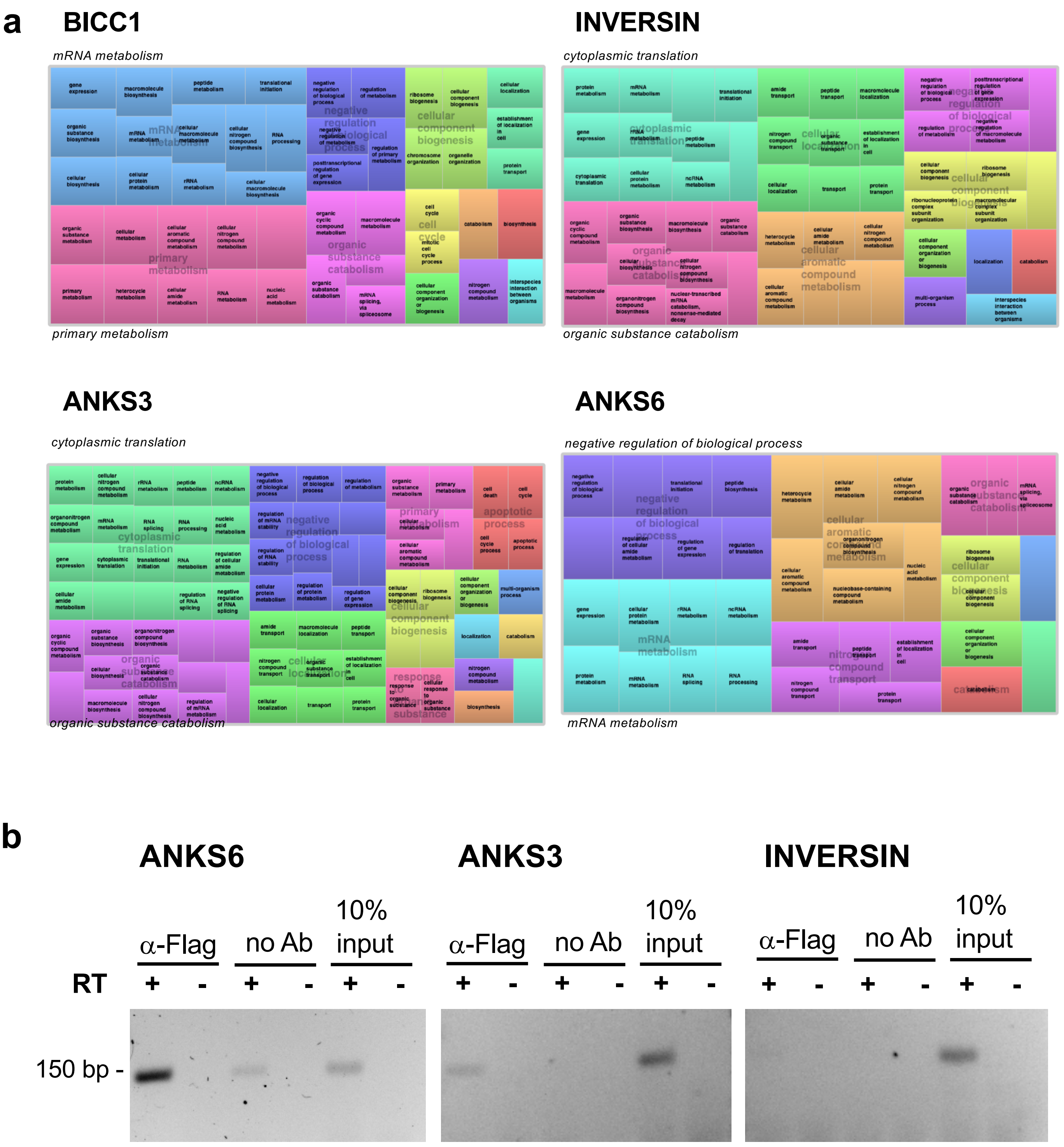

Supplemental Figure 8

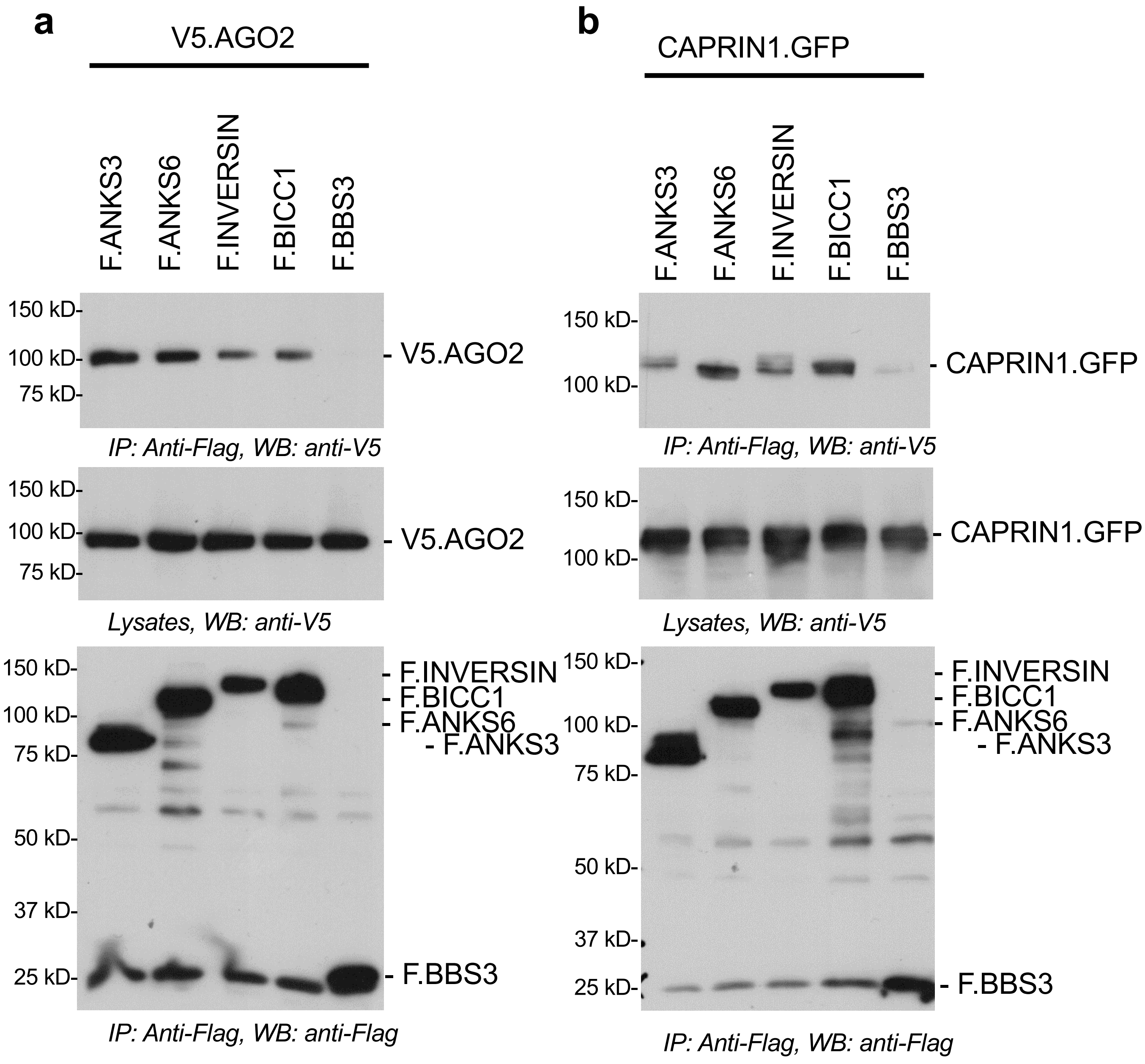

Supplemental Figure 9

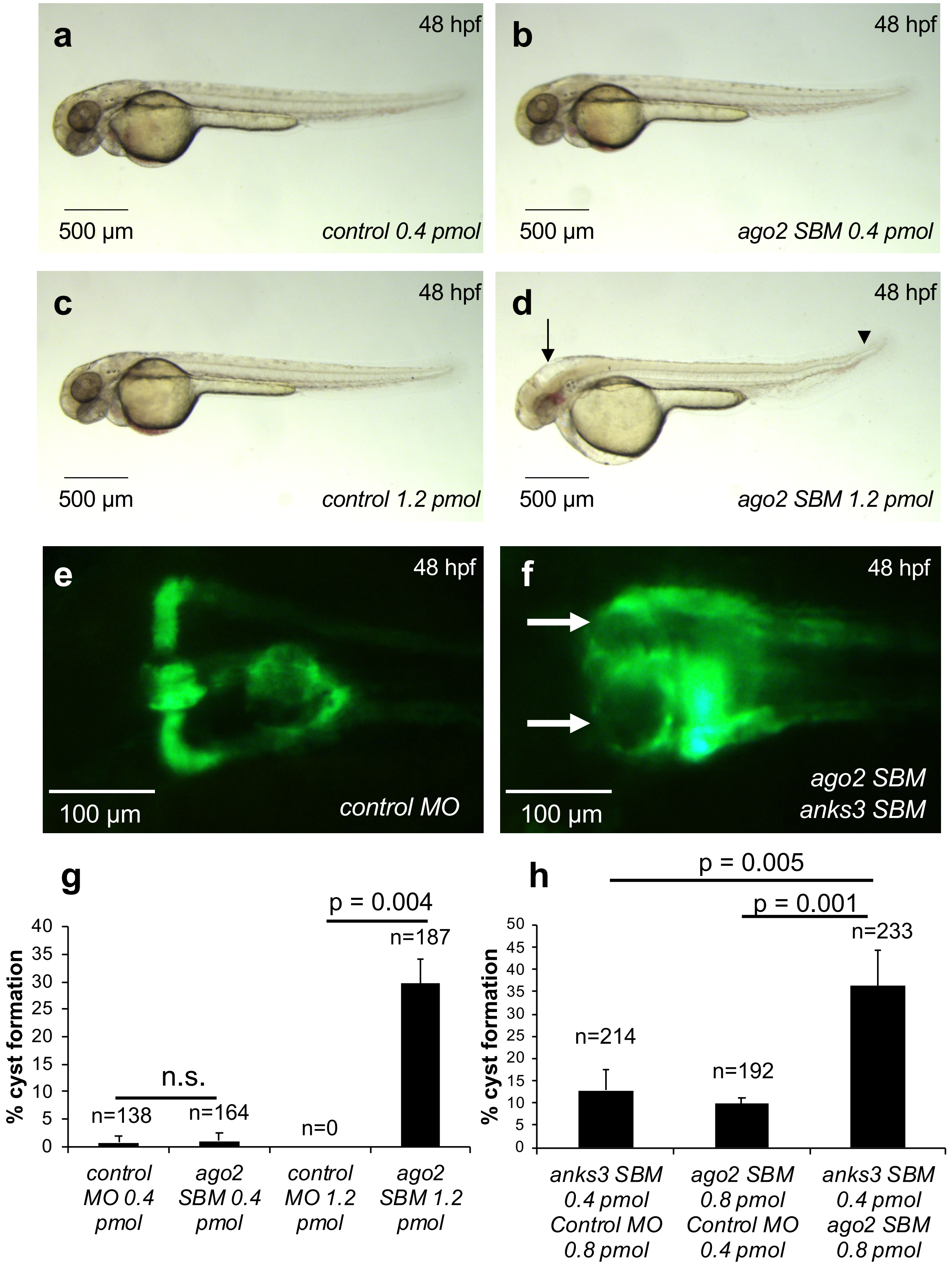

Supplemental Figure 10

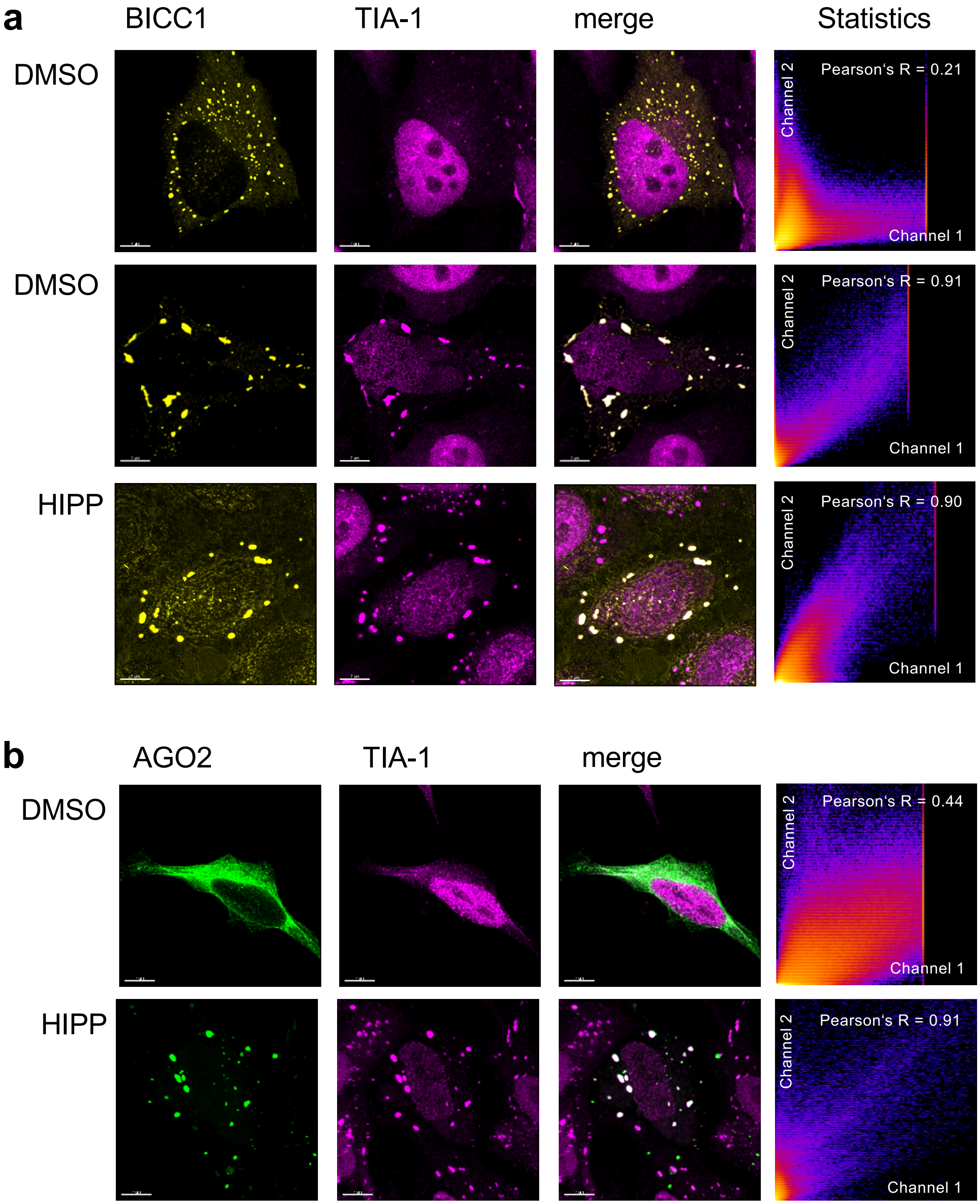

Supplemental Figure 11

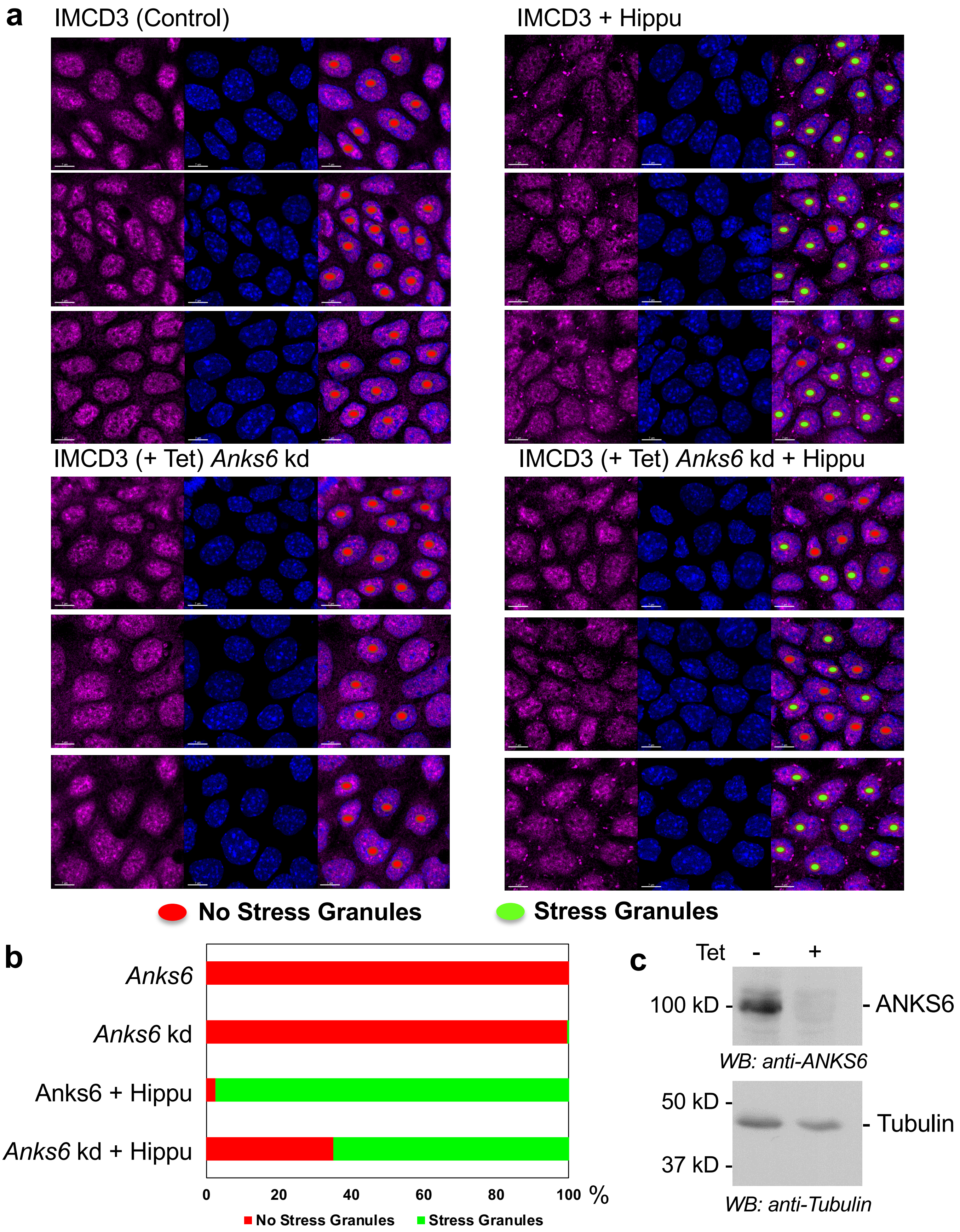

Supplemental Figure 12

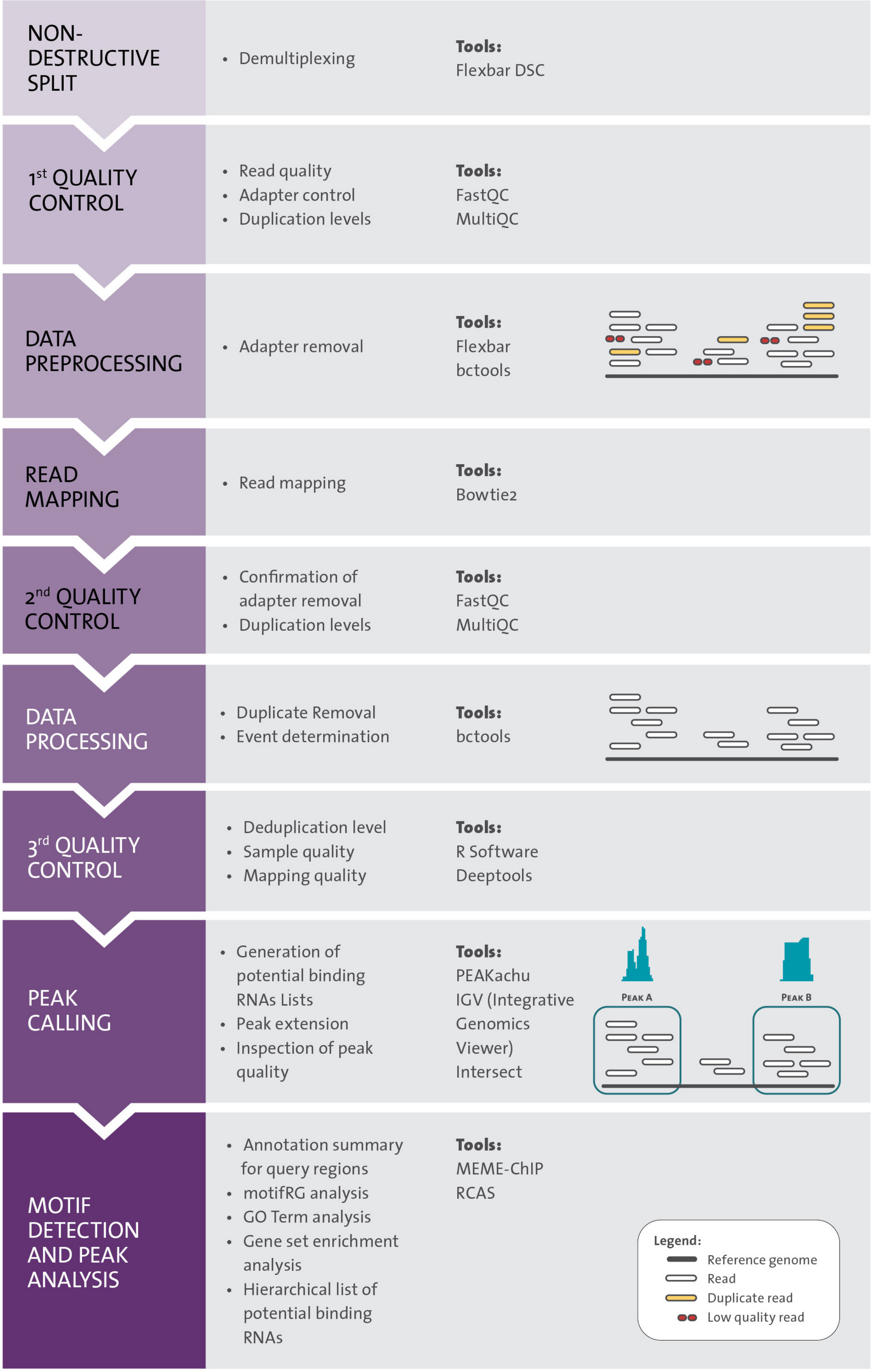

Supplemental Figure 13

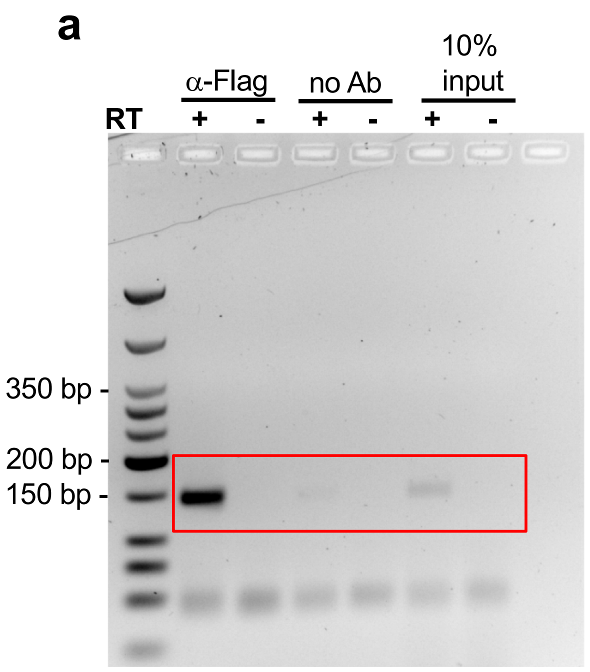

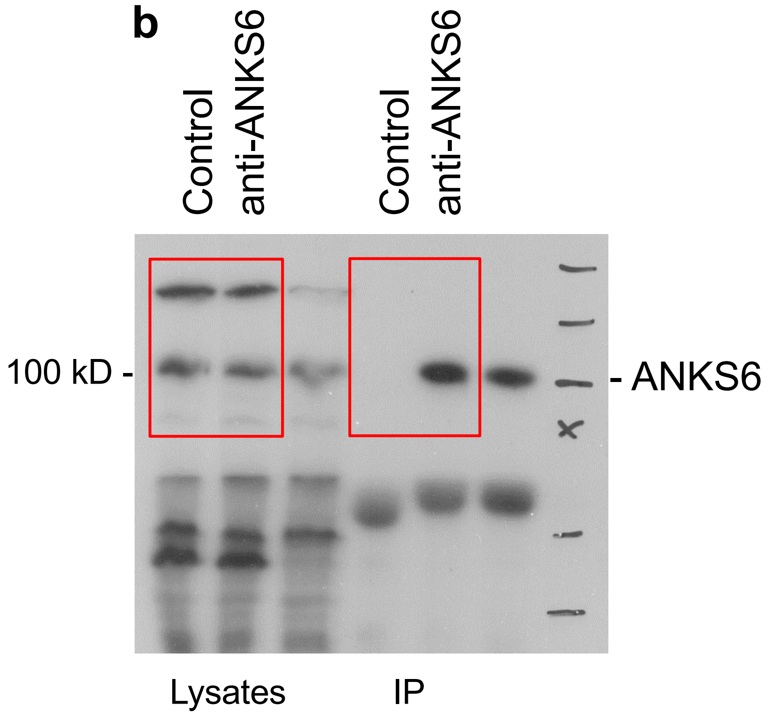

Supplemental Figure 13 (cont.)

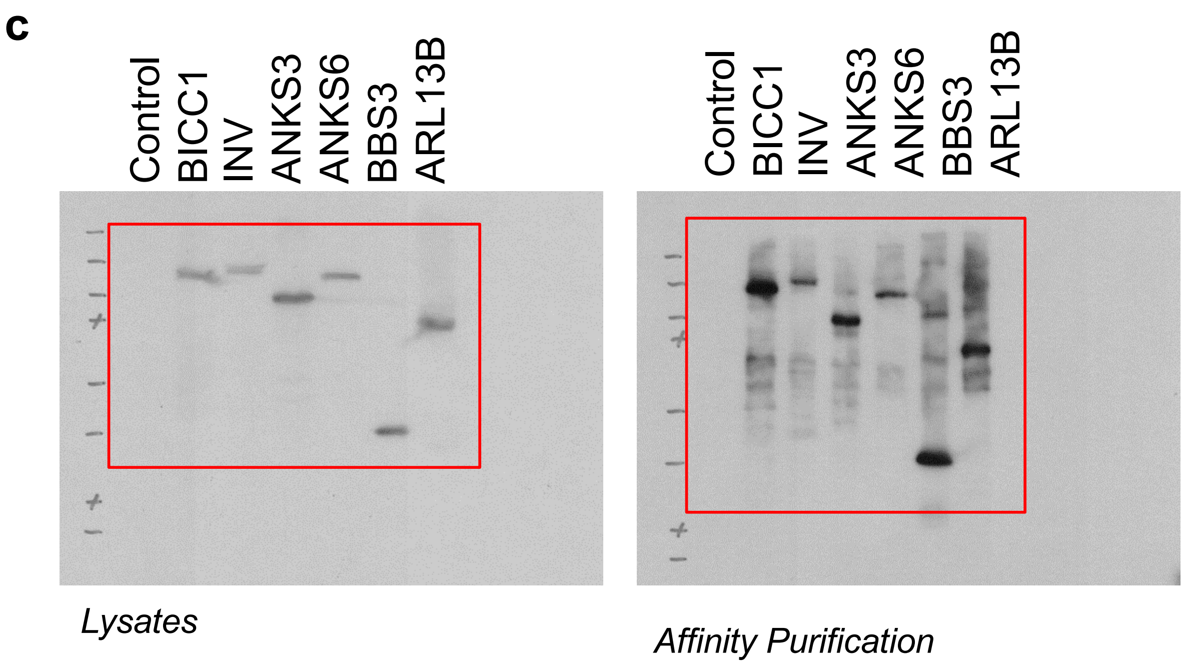

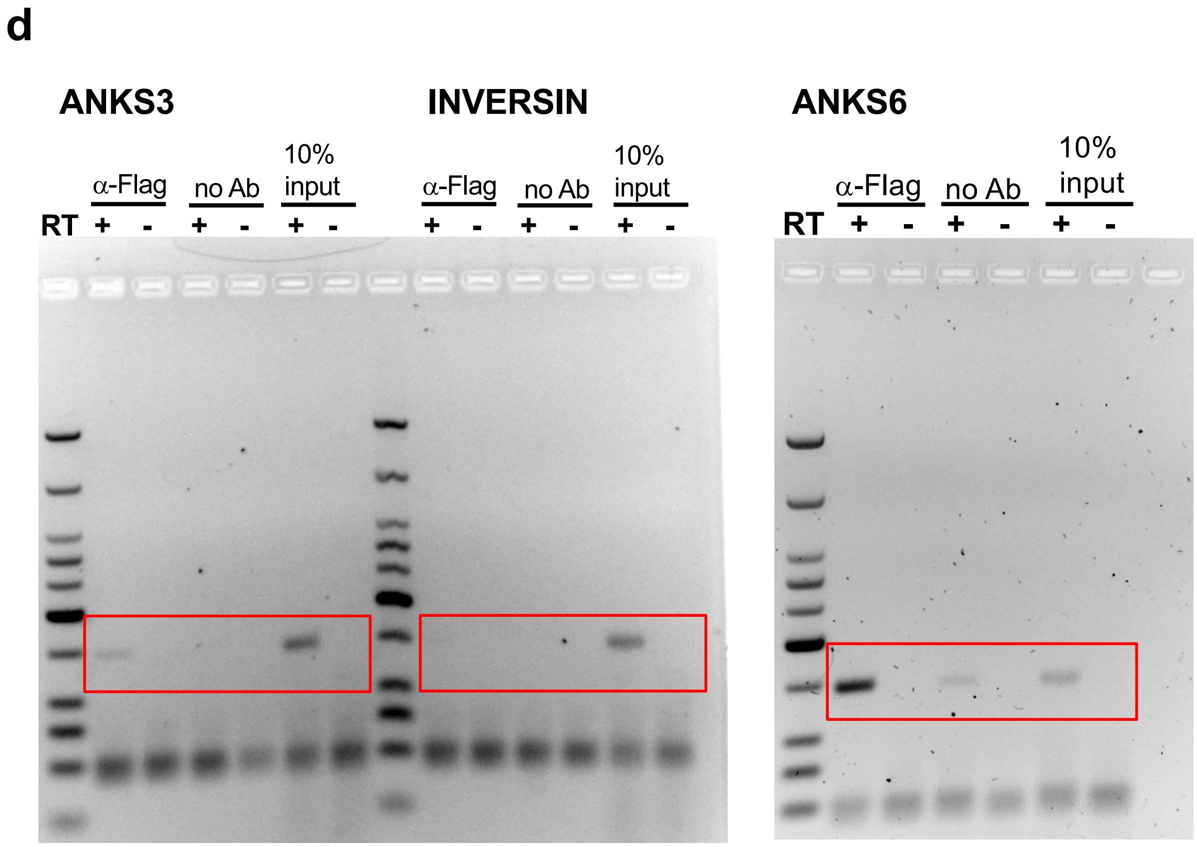

Supplemental Figure 13 (cont.)

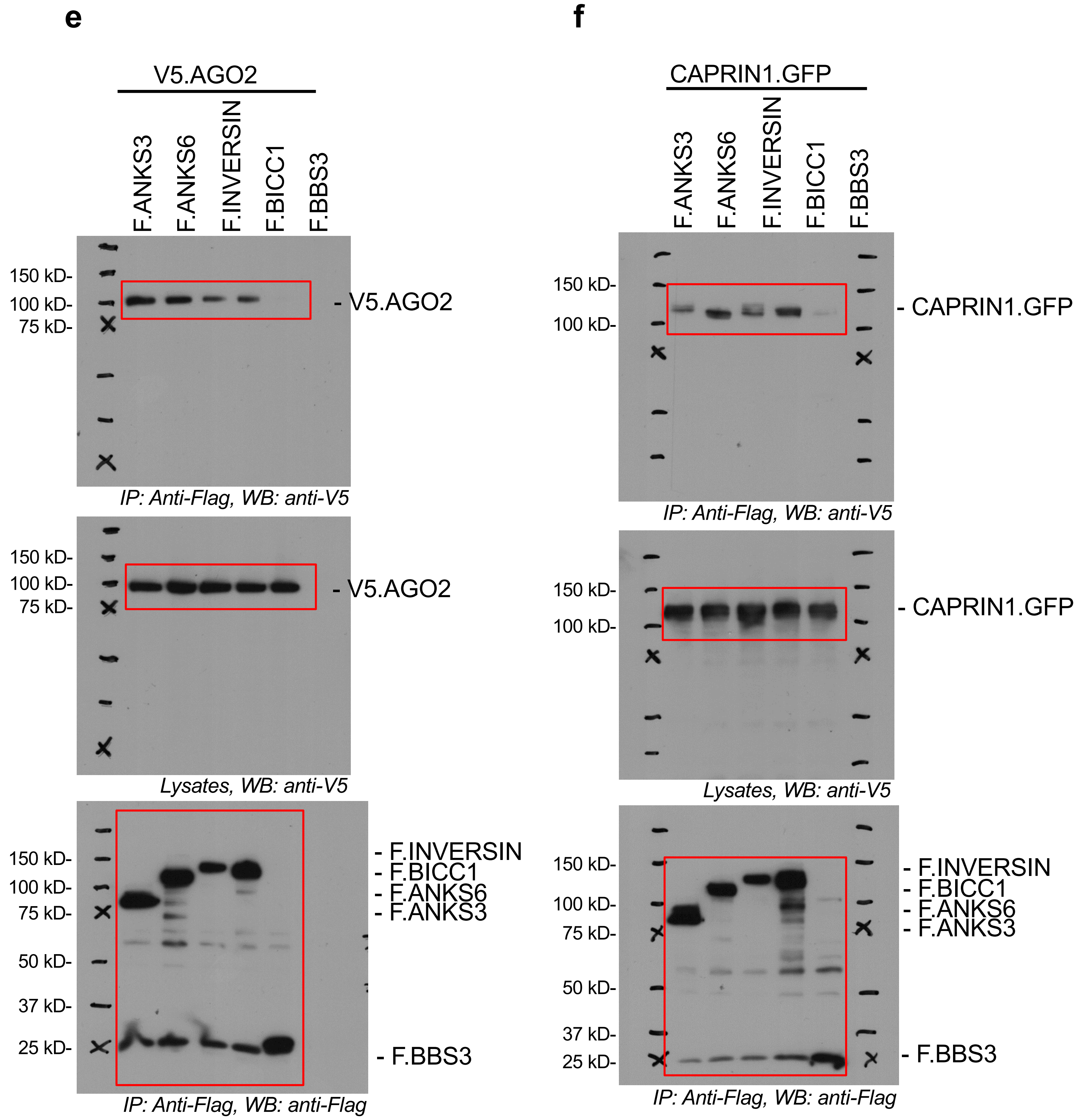

Supplemental Figure 13 (cont.)

g

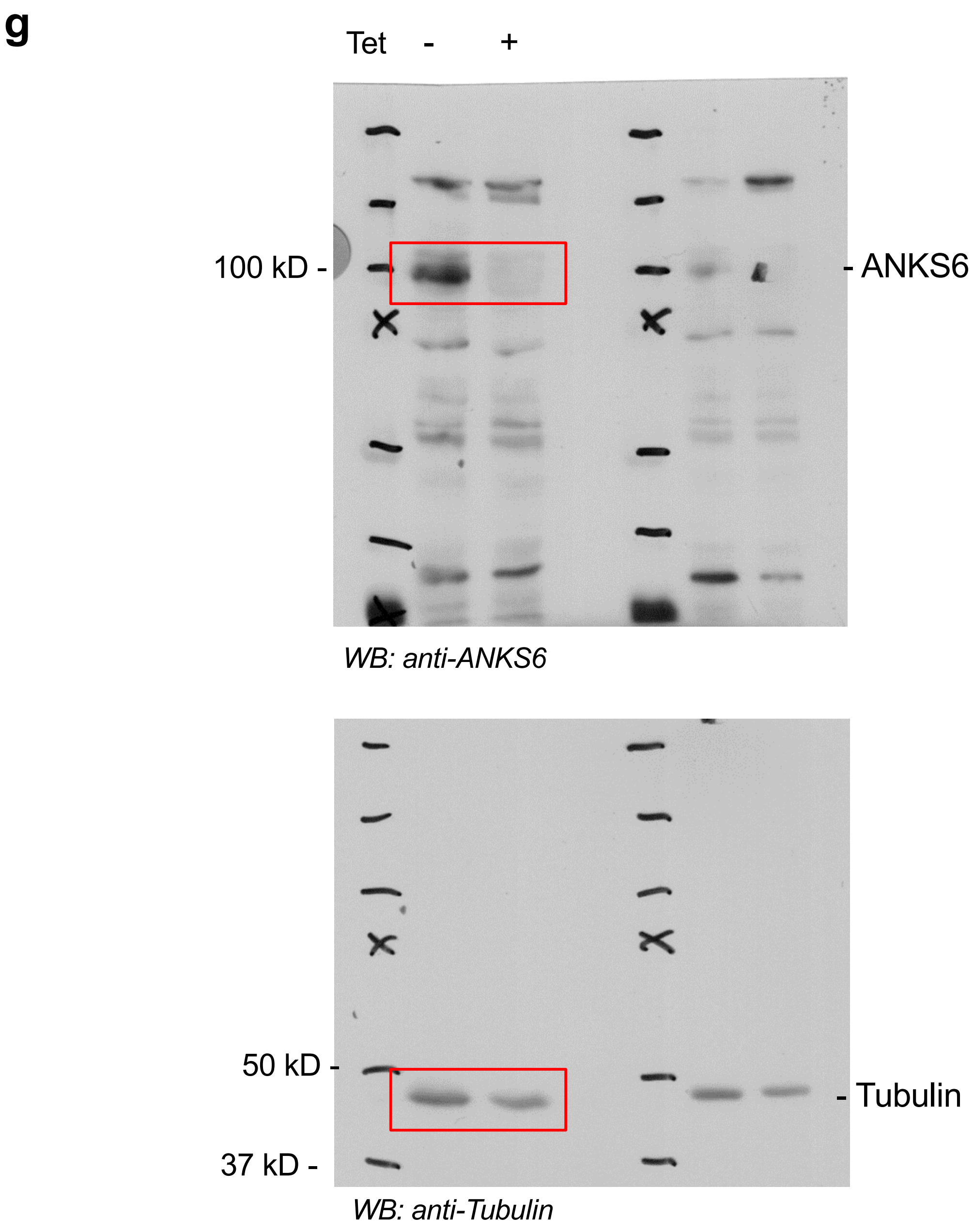

Supplement: Supplementary file 2 — Supplementary Figures. [file 41598_2020_72905_MOESM2_ESM.pdf]
